# Computational Modeling: Human Dynamic Model

**DOI:** 10.3389/fnbot.2021.723428

**Published:** 2021-09-24

**Authors:** Lijia Liu, Joseph L. Cooper, Dana H. Ballard

**Affiliations:** ^1^Department of Computer Science, The University of Texas at Austin, Austin, TX, United States; ^2^Google Inc., Mountain View, CA, United States

**Keywords:** dynamic modeling, motor control, kinematic representation, movement costs, human movement simulation

## Abstract

Improvements in quantitative measurements of human physical activity are proving extraordinarily useful for studying the underlying musculoskeletal system. Dynamic models of human movement support clinical efforts to analyze, rehabilitate injuries. They are also used in biomechanics to understand and diagnose motor pathologies, find new motor strategies that decrease the risk of injury, and predict potential problems from a particular procedure. In addition, they provide valuable constraints for understanding neural circuits. This paper describes a physics-based movement analysis method for analyzing and simulating bipedal humanoid movements. The model includes the major body segments and joints to report human movements' energetic components. Its 48 degrees of freedom strike a balance between very detailed models that include muscle models and straightforward two-dimensional models. It has sufficient accuracy to analyze and synthesize movements captured in real-time interactive applications, such as psychophysics experiments using virtual reality or human-in-the-loop teleoperation of a simulated robotic system. The dynamic model is fast and robust while still providing results sufficiently accurate to be used to animate a humanoid character. It can also estimate internal joint forces used during a movement to create effort-contingent stimuli and support controlled experiments to measure the dynamics generating human behaviors systematically. The paper describes the innovative features that allow the model to integrate its dynamic equations accurately and illustrates its performance and accuracy with demonstrations. The model has a two-foot stance ability, capable of generating results comparable with an experiment done with subjects, and illustrates the uncontrolled manifold concept. Additionally, the model's facility to capture large energetic databases opens new possibilities for theorizing as to human movement function. The model is freely available.

## 1. Introduction

The complexity of human motion was first dramatically captured via the Muybridge high-speed photographs (Muybridge, 1887; Andriacchi and Alexander, 2000; Wolpert and Landy, 2012) which spawned several different analysis techniques in different disciplines. Visualization first used keyframing techniques, but later sophisticated models were used in advanced rendering for computer graphics (e.g., Zordan and Hodgins, 2002). The early cognitive analyses of human behavior (Badler et al., 1993) focused on human motion in problem-solving, using an essentially logical approach. In robotics, insights have been obtained by building physical systems directly (Ijspeert et al., 2007) that straddle the boundary between humans and robotics that have shed light on human design. However, these efforts are characteristically specialized. In another development, machine learning techniques have been introduced for use in analyzing animal-like motion (Schulman et al., 2016).

At the neural level, the brain's control of movement utilizes an array of specialized subsystems. One is the vast cortical memory of movement plans. Others are the sequencing via the Basal Ganglia and mediation of input and output via the Thalamus. The details of how they coordinate (Callahan et al., 2013) are being continually worked out, but there are many open problems (Loeb and Tsianos, 2015). Given this incompleteness, a research choice can be to search for descriptions at more abstract levels.

Since movements have to respect the dynamics of the musculoskeletal system, one approach is to take these systems for granted and start with measurements of motion and work backward. However, modeling all the complexity of the human musculoskeletal system can be challenging, with over 300 degrees of freedom and 650 muscles controlling a complex interconnected skeletal system. Nonetheless, there are many modeling approaches with a dynamics focus, which can be introduced approximately chronologically.

A straightforward approach is to build a humanoid dynamic model. This method is a valuable way to imitate human movements, and it is widely used in biomedical engineering due to its compliance with real-world physical rules. However, it is not without limitations: (1) it is too difficult to model and control a complex system like the whole human body. (2) it is complicated to represent “kinematic loops,” such as postures with both feet on the ground. (3) for large systems, the equations of motion in nested, rotating reference frames become very complex, making them demanding to approximate well. As a result, due to the complexity of the direct modeling method for large complex systems, the significant portion of the studies choose the simplification of two-dimensional models that constrain body motions to the sagittal plane.

For example, some studies build a two-dimensional dynamic bipedal robot by modeling the whole body with a skeleton of rigid segments connected with joints. However, those method's simplifications to human bodies limit studies to simple single-behavior human movements. The simplest bipedal robot uses three links to represent the torso and two legs in the sagittal plane (Lee, 1988; Čelikovskỳ and Anderle, 2018). Nonetheless, there have some extensions. Five-link biped robots use two links to represent each leg (Mu and Wu, 2003, 2004; Krishchenko et al., 2007), and seven-link biped robots further extend it by adding feet (Mousavi and Bagheri, 2007; Bajrami and Murturi, 2017). Furthermore, those methods also make many assumptions in studying locomotion. For example, most researchers assume an instantaneous exchange of biped support occurs when the swing leg contacts the ground. In this way, as robot manipulators, the biped foot support can be considered a successive open loop of kinematic chain from the support point to the free ends. There have been some recent three-dimensional model improvements to the two-dimensional biped robot models (Grizzle et al., 2010; Khusainov et al., 2016; Bailly et al., 2021). However, they are still not sophisticated compared with a real human body.

To get beyond the disadvantages of these simple models, a major way forward is to incorporate more detailed musculoskeletal models (Durandau et al., 2017; Lee et al., 2019). Among them, the most advanced and sophisticated one is OpenSim (Delp et al., 2007; Seth et al., 2011; Rajagopal et al., 2016; Dembia et al., 2020), which allows modeling large systems including detail to the level of attached muscles. OpenSim is a significant advance by providing a modeling level that includes muscle contractions, which are essential in shaping movement dynamics. However, this level works best in fitting contraction data. It can be challenging to solve desired muscle co-contractions from a movement kinematic plan because those equations are non-linear and normally under-constrained.

To avoid this complexity, many studies that need such generating capability choose to eschew muscle components and focus on simpler dynamic models at the level of inertias and joint torques and model abstract versions of the human system that still use multiple degrees of freedom but summarize detailed dynamics via joint torques. For example, co-contraction can use a simple Hill model (Blümel et al., 2012). These alternative methods of dynamics computation of such multi-jointed systems have also experienced significant advances (OpenSim can also be used in this way). The foremost of these is to use a kinematic plan to integrate the dynamic equations directly. Several dynamic libraries were designed for this purpose, such as MuJoCo^1^, Bullet^2^, Havok^3^, Open Dynamic Engine(ODE)^4^, and PhysX^5^. An evaluation of these dynamic libraries by Erez et al. (2015) found them roughly comparable in capability. However, the focus of these systems is on a physics engine with the expectation that the users will program their own applications.

In contrast to previous studies, our Human Dynamic Model (HDM) system is a complete model that is readily available ^6^,^7^. It is built on top of the physics engine ODE, the most commonly used dynamic library in the robotic area. Our 48 degree-of-freedom HDM focuses on individual human movement modeling and applies a direct dynamics integration method (Cooper and Ballard, 2012; Johnson and Ballard, 2014) to extract torques from motion data using a novel unifying spring constraint formalism.

One of the advantages of the HDM system is that the simulation's costs can be recovered. At each frame, the instantaneous power is computed from the net joint torque and joint angular velocity. The work performed at each joint is determined by numerically integrating the instantaneous powers over the entire tracing task. In this way, given motion capture data, we can compute the mechanical cost without building a humanoid biped robot with motion equations. Note that it is common to use mechanical measures of work to indicate cost instead of metabolic energy consumption (Burdett et al., 1983). The “energetic cost” mentioned in the following sections means the mechanical cost.

The focus of the paper is to describe the HDM simulator as a practical laboratory instrument and detailed demonstrations that illustrate the model's capabilities. The paper is organized as follows: first, the methods section starts with an introduction to the model topology and then elaborates on the use of the motion markers to capture the model's joint torques, which allows the estimation of energetic costs. Next, the results section describes several tests taken to measure the performance of the HDM system. After that, the discussion section highlights the features and issues of the HDM and points out one of the potential applications of the model, which is its use to capture large amounts of analyzed movement data. Finally, the Supplementary Material includes two appendices that represent the essential low-level implementation of the model, which is the integration steps of the dynamics equations.

## 2. Methods—Model Design and Operation

This section provides a comprehensive exposition of building the human dynamic model from high level. Low level details of building the HDM and the derivation of the mathematics underlying the physics simulation are presented separately in the Appendix.

### 2.1. Body Structural Details

**Figure 1:**
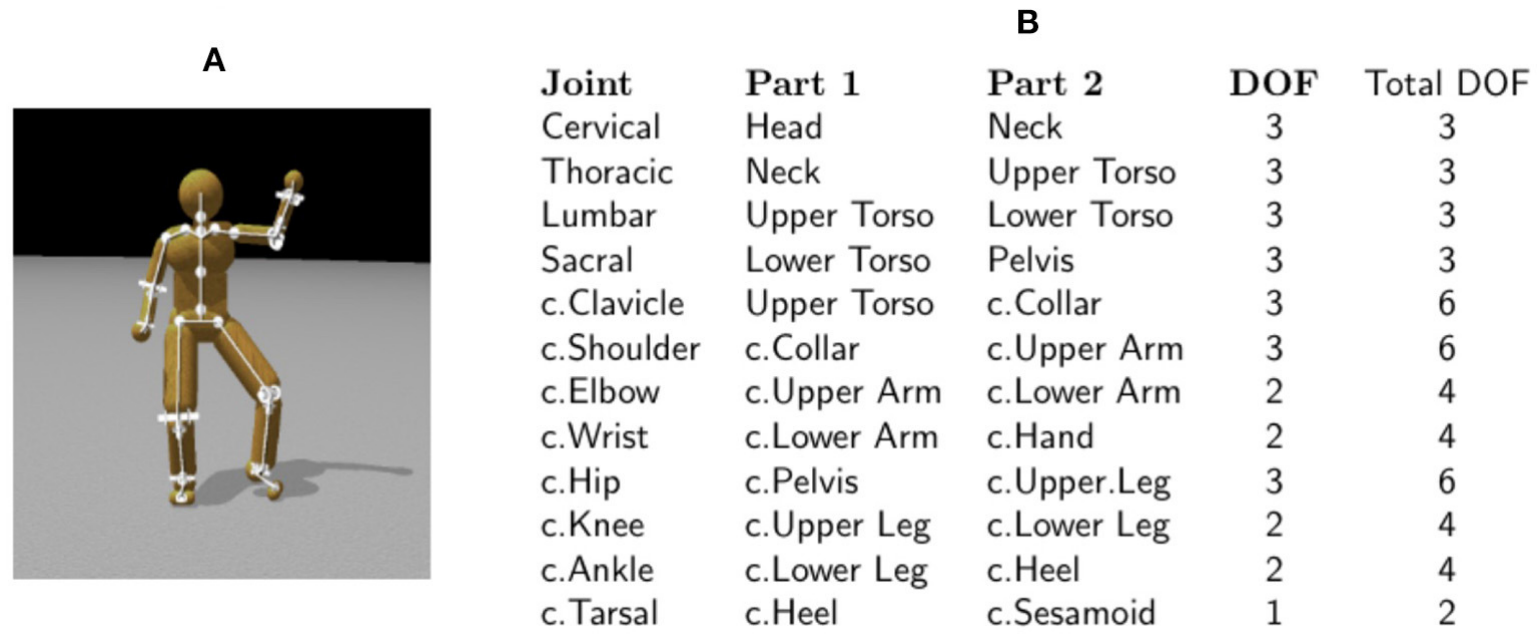
The 48 degree of freedom model. **(A)** The skeleton of a character model: 21 body segments connected by 20 joints. **(B)** A summary of the joints used in the model. c., chiral: there are two of each of these joints (left and right). Four ball-and-socket joints connect five body-segments along the spine from the head to the waist. Ball-and-socket joints are also used at the collar-bone, shoulder, and hip. Universal joints are used at the elbows, wrists, knees, and ankles. Hinge joints connect the toes to the heels. All joints limit the range of motion to angles plausible for human movement. Our model assumes that joint DOFs summarize the effects of component muscles.

Our techniques use a simulated model of the human whose movement is analyzed. The first order of business is to build a physical model capable of representing human movements, of which the accuracy influences the outcome of the analysis. Figure 1 shows the body segments and topology of the model. The humanoid model is a collection of rigid bodies connected by joints. Each joint connects two rigid bodies with anchor points (center of rotation), defined in the reference frame of both bodies. The body dimensions (bone lengths) of the character model were determined based on motion capture data.

### 2.2. Data Fitting

**Figure 2:**
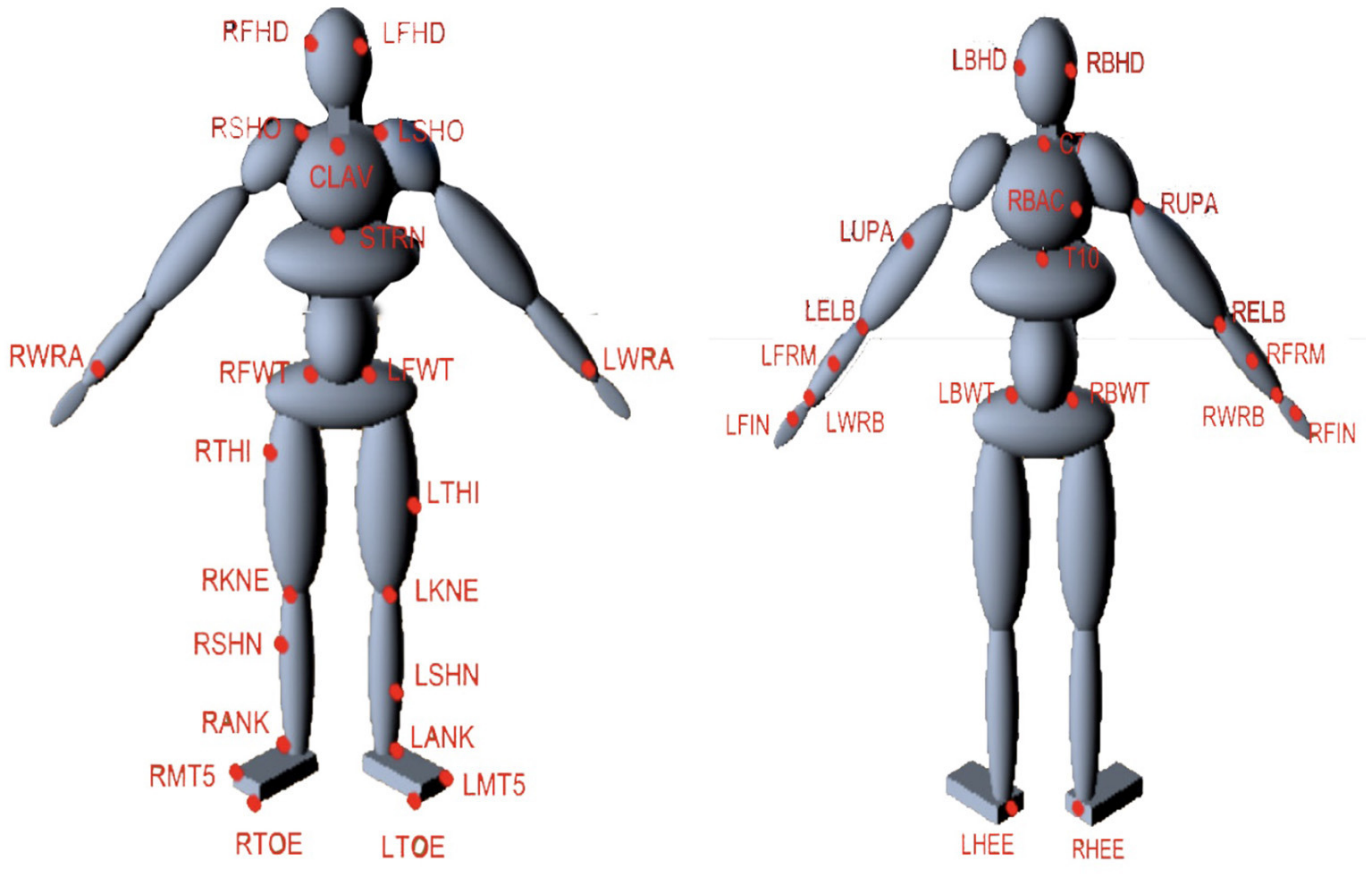
Marker arrangement on the HDM. The suit contains 51 markers as shown by the LEDs in total but only 41 are used in the model, e.g., Markers that are unused are present on the fingers. Markers can easily assigned to specific model segments. For example, the markers of RBHD, RFHD, LFHD, and LBHD are assigned to Head segment while the markers of RBWT, RFWT, LFWT, and LBWT belong to Pelvis segment.

The technique for fitting a human model to motion capture data begins with a character model that serves as a template, Figure 1 presenting the number of body segments and topology of the model. We further assign all labeled markers used in motion capture to specific model segments. It is straightforward to derive these using techniques such as in Kirk et al. (2005) and De Aguiar et al. (2006). However, manually assigning markers is also not complicated because the motion capture suit typically puts markers on the same body segments (Figure 2), even if they are in slightly different places or the body segments have different dimensions.

We present a method in S2 Appendix section, for using marker data to help determine the dimensions of the model segments and where markers attach to the model. Although this method could easily be automated, in practice, the research did not rely on very many different models, so the system uses a mechanism for relaxing the marker attachment points and joint anchors with the click of a button in the graphical user interface (**Figure 5**). With a new data set, a handful of iterations proved sufficient to produce a reasonable model with marker attachments that fit the data well enough for further analysis. This algorithm does not address joint limits on a range of motion. These can also be learned (Tournier et al., 2009), but in our case, the range of motion for each joint is seta priori. After determining segment lengths, we set other segment dimensions as appropriate to fit against the markers. Mass properties for each segment assume uniform density by volume.

Given motion capture data of a subject, the model is fit to the subject's dimensions, and joint-range-of-motion is constrained to approximate the subject's flexibility. Additionally, the model segments have inertial matrix properties. The initial mass assignment to each segment assumes a uniform density of water ($1,000\frac{kg}{m^{3}}$) for the volume associated with each rigid body. The mass assignment should be modified to match that of a specific subject roughly. The increased fidelity required for individual subjects in clinical biomechanics research would employ more sophisticated techniques to better approximate mass distribution in the model. However, interestingly the experimental results discussed above show that even this low fidelity model is sufficient to produce high-quality data that compares favorably with data gathered from independent sensors.

### 2.3. Pose Fitting

Having addressed the issues in attaching the model to motion capture data, we turn to the construction of its capability of representing human movements. Various commercial packages provide different methods for converting marker trajectories into sequences of body poses, but they can be time-consuming, expensive, or difficult to use. This section describes an approach related to Demircan et al. (2008) and Zordan and Van Der Horst (2003) that is free, fast, uses intuitive parameters, and allows the user to fit markers to whatever model they wish.

The method uses the physics engine to constrain a character model to fit marker data and other constraints. Markers are modeled as infinitely massed points attached to the character model. Given a frame of marker data, the position and orientation of all body segments can be found by balancing internal joint targets and external marker data. From the global position and orientation of the different body segments, it becomes simple to compute relative orientations (joint angles).

**Figure 3:**
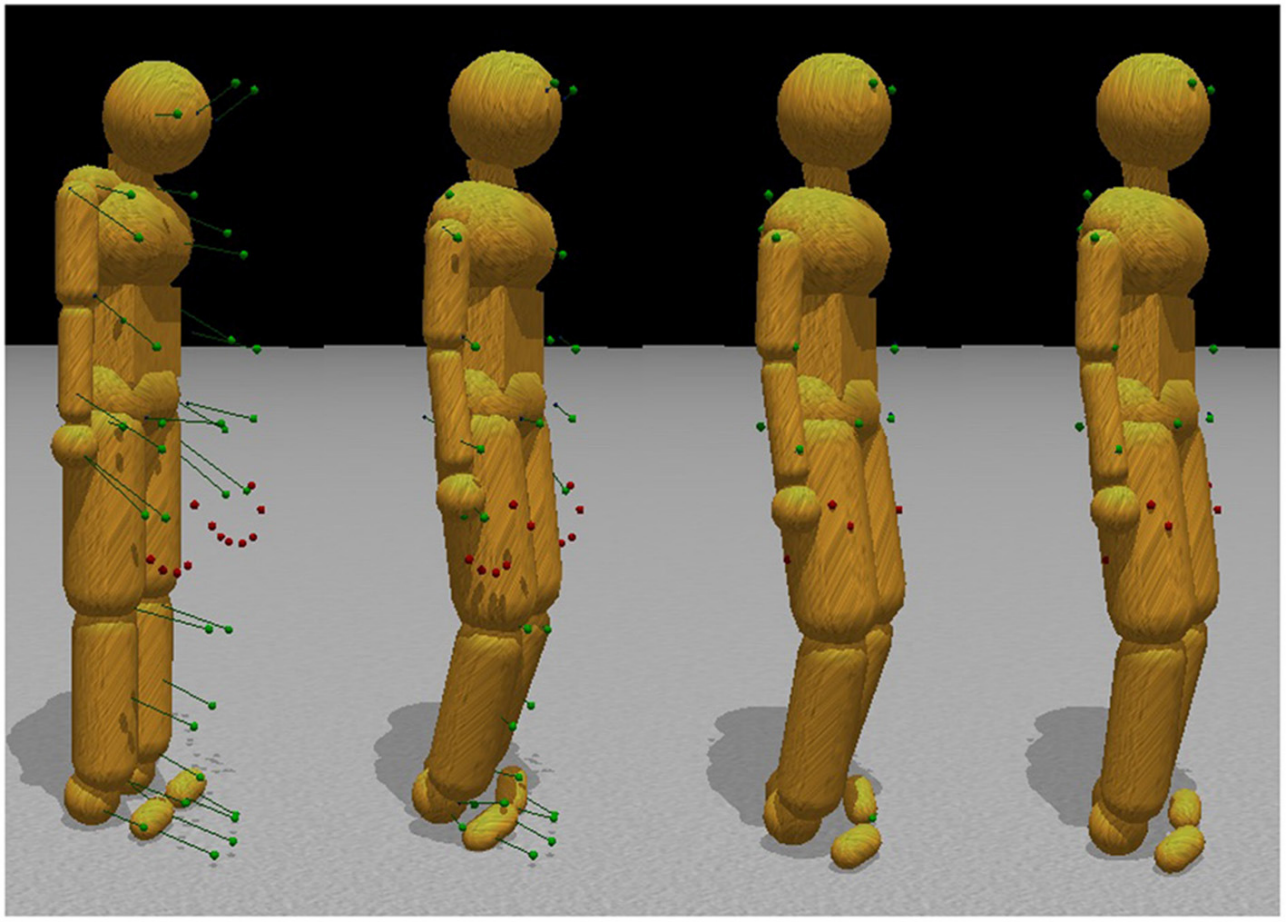
Pose fitting. Initially, the motion capture data points are in a very different configuration than the initial stance of the model. To find the appropriate correspondences, simulated markers attach to the humanoid model through ball-and-socket joints and pull the body parts into place, subject to model joint constraints. The left to right sequence in the figure shows the body targets being gradually reconciled with the external markers.

The internal degrees of freedom are limited by the range of motion constraints, e.g., the elbows and knees cannot bend backward. All other joints have similar range-of-motion limitations based on the subject's flexibility. Furthermore, each joint is set to have a “target state,” a preferred relative orientation between its connected bodies. These preferences can be thought of as “muscle stiffnesses” and are modeled as weak constraints with limited force. Joint limits and stiffness serve as a prior over possible poses so that in the absence of any marker data, the model still takes on a pose. Consequently, every internal degree of freedom is constrained to some degree. These constraints hold the model together and give it a default pose. Next, in a pivotal step shown in Figure 3, marker data pull the model from the default pose into a new pose. Each marker is connected to a body segment using a ball-and-socket joint constraint for a given frame of motion capture data. A total of 41 markers, which do not contribute any degrees of freedom because of their infinite mass, attach to the character model, adding additional 3 × 41 = 123 constraints.

Finally, collisions between the ground and the feet also influence the model pose. Each foot can form up to three contact points with the ground. Inequality constraints at these points prevent penetration with the ground. When both feet are firmly on the ground, all markers are actively pulling the body into a pose, all joints are holding the body together, and joint limits and stiffness are biasing the relative orientation of the bodies. The experiments described above show that the model can simulate the ground force correctly.

This approach is intuitive: attach markers to the model with springs and drag the body along. The parameter, tunable for each constraint, known in ODE as the constraint force mixing parameter (CFM)^8^, allows a constraint to slip proportional to the amount of force required to maintain the constraint. We use a CFM value of 1 × 10^−5^ for the regular internal body joints and contact constraints while using 1 × 10^−4^ for the constraints between markers and body parts. Both of these values represent very stiff springs, although they are different by order of magnitude. This stiffness stabilizes the simulation by allowing the markers to stretch slightly from their mapped locations if the marker constraints are not compatible with the character model. Figure 3 shows that when the markers move, the constraints drag the character along with them.

### 2.4. Inverse Dynamics

It can be useful to know the torques to apply at each joint or the required effort to accomplish a particular movement. Given a kinematic sequence of body poses, the physics engine ODE can archive the computation with minimal effort. Given constraints like each joint's angular velocity, it can correctly compute the desired torques/forces measurements.

The process is straightforward. Given the current joint angle and the desired joint angle for the next frame, the body parts' relative angular velocity is constrained to achieve the target orientation on the next frame. Contact constraints are necessary to prevent ground surface penetration as well. The ODE physics library handles the constraints and solves the torques and forces that satisfy each constraint in the process.

For computing inverse dynamics, the first step is to initialize the model to a starting dynamic state. The initial state can be found from the first and second frames of kinematic pose data. The model pose is set by using the second frame of data, and the initial linear and angular velocity of each joint is computed by taking the finite difference between the two frames (and dividing by the time-step). Computing velocity through finite differences is appropriate for a physics engine using first-order semi-implicit Euler integration. After that, continuously find the torques between two consecutive frames of pose data using the finite difference between poses to compute angular velocities that will move the model from the current to the next pose.

Differentiating again, this time between the current and future velocity gives a target acceleration that constraints the model. The primary difference between this step and the previously discussed method for finding pose from marker data is that no marker constraints are dragging the body into place. The internal dynamic constraints drive the model toward a target pose on each frame instead of toward a “default” pose. Because there are fewer constraints in play, stiffer muscle forces are used, but the absolute forces the muscles can apply are limited to prevent muscle forces from being unreasonably large. Again, we can use the relative spring stiffness to express confidence in the measurements in this case. We use very stiff springs (CFM = 10^−10^) to keep the model segments together. We use looser constraints to keep the feet from penetrating the ground (CFM = 10^−5^) and to constrain the model to adopt the appropriate pose (CFM = 10^−8^).

#### 2.4.1. Residual Torques/Forces

The torque calculation by the HDM is ideal in the sense of solving the inverse dynamic equations. The inverse dynamics uses measured kinematics and external forces to calculate net joint torques in a rigid body-linked segment model (van der Kooij et al., 2005). However, discrepancies between the dynamic forces of the model and the kinematic of the reality make it so that the dynamic model falls over unless action is taken to stabilize it. Therefore, there needs a corrective system for unexpected errors in practice. In the human system, there are multiple corrective systems based on vision, proprioception, and the vestibular system. Such corrective systems have been extensively studied (e.g., van der Kooij et al., 2005; Sentis et al., 2010; Welch and Ting, 2014).

In classical inverse dynamic areas, a common way to compensate for this problem is by introducing “residual forces and torques” (van der Kooij et al., 2005). In the HDM, a 6 degree of freedom joint between the waist segment and the global frame generates the external forces. A weak, limited spring constrains the waist segment to achieve its recorded pose relative to the global frame. The experiments show that attaching the external constraint to the head or the feet has little noticeable difference. The non-realistic external forces (residuals) account for noise as well as discrepancies between the model and the human generating the data. In particular, differences in how the feet interact with the ground cause errors in our analysis. In most cases, it is only necessary to constrain two of the six angular degrees of freedom (pitch and roll), leaving the other four external degrees of freedom disabled. The two angular constraints keep the body from falling over but allow it to move about through simulated ground interactions.

**Figure 4:**
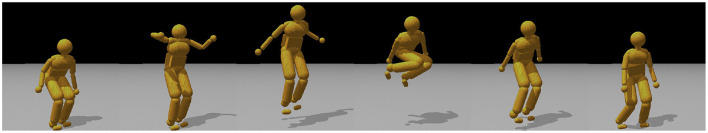
Model capability illustration. A complex jump sequence reproduced with physics-engine-based inverse dynamics using recorded motion capture data from a human subject. The recreated jump height is achieved completely from ground forces, augmented with small residual torques (≤ 100 Nm), allowing the model to maintain balance.

The stabilization system completes the model. It can be implemented in parallel, with the control used to stabilize the residual necessary to balance. With this included, The simulation can reproduce highly dynamic motions (e.g., see Figure 4), which shows a jumping sequence made originally by a human subject and recreated using the torques computed by the inverse dynamics model.

### 2.5. Method Summary

We construct a dynamic model for each human subject and force the model to follow the subject's motion capture data, which leads directly to the recovery of joint angles. Our algorithm constrains the dynamic model to track these angles and consequently can estimate the correct joint torques. This concept was originally demonstrated in two dimensions for human walking by Faure et al. (1997). We have extended the method to the significantly more demanding case of 48 DOFs in three dimensions and arbitrary posture changes. Figure 1 lists the body segments. The dimensions of each segment are matched those of an individual subject. The principal difficulty is that the constraints in the high DOF 3D model present many delicate numerical issues for the ODE solver that need to be addressed (Cooper and Ballard, 2012). Currently, the dynamic model does not attempt to model stiffness components, with the consequence that it can only directly recover the net torques at each DOF.

The calculation of mass properties is crucial for simulating rigid body collisions. Mass and inertial are computed using the volume of the body parts with a constant density of $1,000\frac{Kg}{m^{3}}$. The articulations are designed to allow the dynamic model to simulate the majority of human movements. For instance, elbow joints have two DOFs to represent the hinge movements of the elbow as well as the twisting movements of the radius and ulna bones in the arm. Joint angles are also limited to avoid impossible movements, such as reverse bending of the elbows or knees.

For data capture, a subject wears the motion capture suit developed by PhaseSpace. Each LED marker on the suit is mapped to a corresponding point on the model. The markers are then introduced into the physics simulation as kinematic bodies without collision geometry. As a heuristic, each marker is considered infinite mass. Thus, when another dynamic body is attached to the marker through a joint constraint, the dynamic body's trajectory will follow the marker's trajectory completely.

The PhaseSpace motion capture system records 3-dimension positions of specific human body locations over time. When the simulation steps forward, the constraint solver adapts the dynamic model to a state that satisfies the internal joint constraints, the external marker constraints, and other constraints such as ground forces and conservation of momentum. Knowing the kinematics allows the recovery of the dynamics since the joint velocities allow the equations of motion to be inverted. The retrieved forces can be used to generate feed-forward torque profiles for actuating the character.

Utilizing the human dynamic model to analyze human movements includes the following five steps:

1. Motion synthesis step: it represents human motion in terms of motion capture data.
2. Inverse kinematics step: the ODE built-in functions are called to compute the joint angular velocities and joint angles at each frame.
3. Forward kinematics step: human motions are simulated based on the joint angles and angular velocities acquired from the previous step. This step is to check the correctness of recovered kinematic properties.
4. Inverse dynamics step: ODE built-in functions are called to calculate the required joint torques.
5. Forward dynamics step: human motions are simulated based on the computed torques and residual forces. This step is to check the recovered dynamic properties against the original motions.

At each frame, instantaneous power was computed from the product of net joint torque and joint angular velocity. The work performed at each joint was determined by numerically integrating the instantaneous powers over the entire tracing task. In this way, the energy cost of human motions can be computed given motion capture data. The overall idea behind the method for calculating joint torques/angles is straightforward using our implementation in ODE. The mathematics underlying the rigid body simulation software used in our work is explained in the Appendix section.

## 3. Results—HDM Basic Capabilities

**Figure 5:**
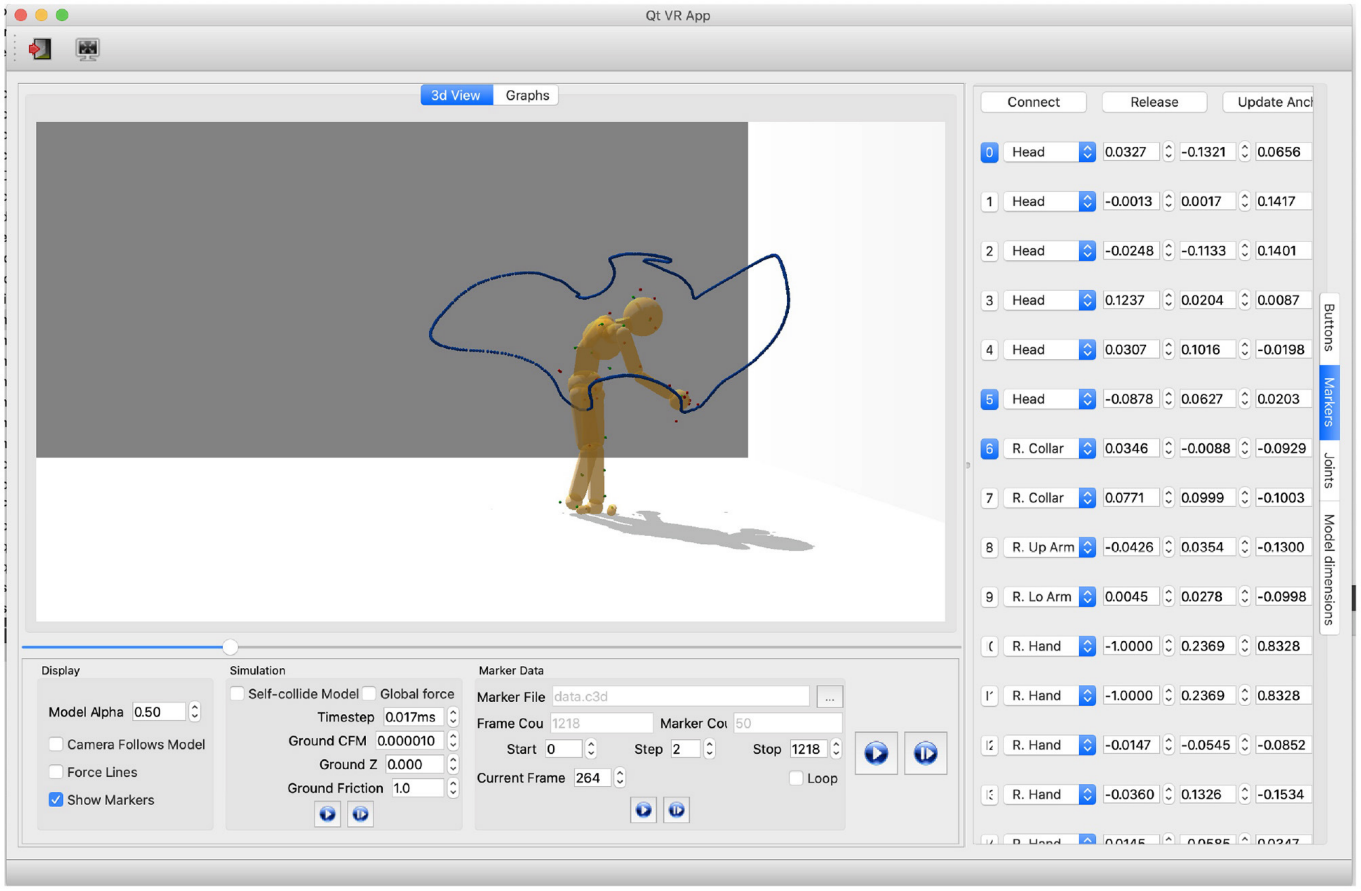
Relevant parameters for analyzing and simulating physically-based movements must be tuned manually. Parameters of the model consist of physical world parameters, joints constraints, and the model's body-marker relative positions. In this depiction shows how users can get the current HMD configurations by clicking the buttons on the rightmost vertical menu. “Marker” is selected, meaning the marker information is shown: (1) The first column represents marker index buttons. Buttons in blue means the corresponding markers are attached to the HDM. Users can attach/detach markers by clicking index buttons. (2) The second column shows body segments where markers are attached. Each spin box is a collective item of all body segment names. Users can use it to change the body-marker attachment relationship. (3) The three-five columns present the marker-body relative positions. Users can modify the values directly using this interface. (4) The “Connect” button and “Release” button on the top are to attach or detach all the markers, respectively. The “Update Anchor” button automatically updates the marker-body relative positions based on the current motion posture.

The HDM is a fast, robust, intuitive, and inexpensive multi-purpose tool for simulating, analyzing, and synthesizing humanoid movement. Figure 5 shows a frame of tracing movements collected from the virtual reality experiment (Liu et al., 2019). Users can specify the configurations of the human dynamic model via a multi-purpose graphical interface^9^ for analyzing movement data captured through interaction with the virtual environment. With this tool, it is possible to manually fit a model to motion capture data, dynamically adjust parameters to test different effects, and visualize the results of kinematic and dynamic analysis. In the particular experiment shown, a subject's fitted model traces a virtual curve to generate a kinematics posture trajectory that allows the cost of the subject's dynamics model to be calculated.

This section focuses on describing the model's capabilities through a series of examples in different settings. Several test experiments provide qualitative and quantitative validation of the physics-based movement analysis techniques described here.

### 3.1. HDM Accuracy

Given that the torque recovery technique will be the basis for our experiments, it is essential to establish its accuracy in absolute terms. A straightforward way to do this is to use a *particular model* to generate joint torque data and then verify that these generating torques can be recovered with sufficient accuracy. To test the model accuracy and noise sensitivity, we first use the PhaseSpace motion capture system to gather the walking data and then let the model simulate the walking motion. To simulate possible sensor errors in the PhaseSpace system, we introduce noise into the simulated marker positions and study recovery accuracy with increasing noise levels.

#### 3.1.1. Model Data Sensitivity Tolerance

Inverse dynamics computations rely on first finding the model's pose. Therefore, given motion capture data, it is essential to synthesize the pose sequence precisely. We used the HDM to synthesize treadmill walking and then compute its accuracy. This study aimed to assess the effect of sensor perturbations on the results and compare the joint angles and torques found with our method to those used to generate marker data. We used an experimental process similar to that employed in Remy and Thelen (2009). In this experiment, both steps were tested by studying eight steps of marker data captured from treadmill walking. The movement lasts a little longer than 4 s, giving us 260 frames of data. For this computation, we used data arbitrarily sampled at 60 Hz.

We used a preliminary pass through the motion capture data to generate synthesized “ground truth” marker, pose, and torque. After using the physics-based inverse kinematics to compute joint angles, we constrained the body to use inverse dynamics to reproduce the joint angles with internal torques (and residual forces at the waist segment). As the model performed the movement, we recorded the global position of the marker attachment points. We also recorded the forces used and the resulting joint angles. Thus we had synthetic “ground truth” data directly from the model. The model is capable of falling over, when it did not, we recorded the test as a success.

**Figure 6:**
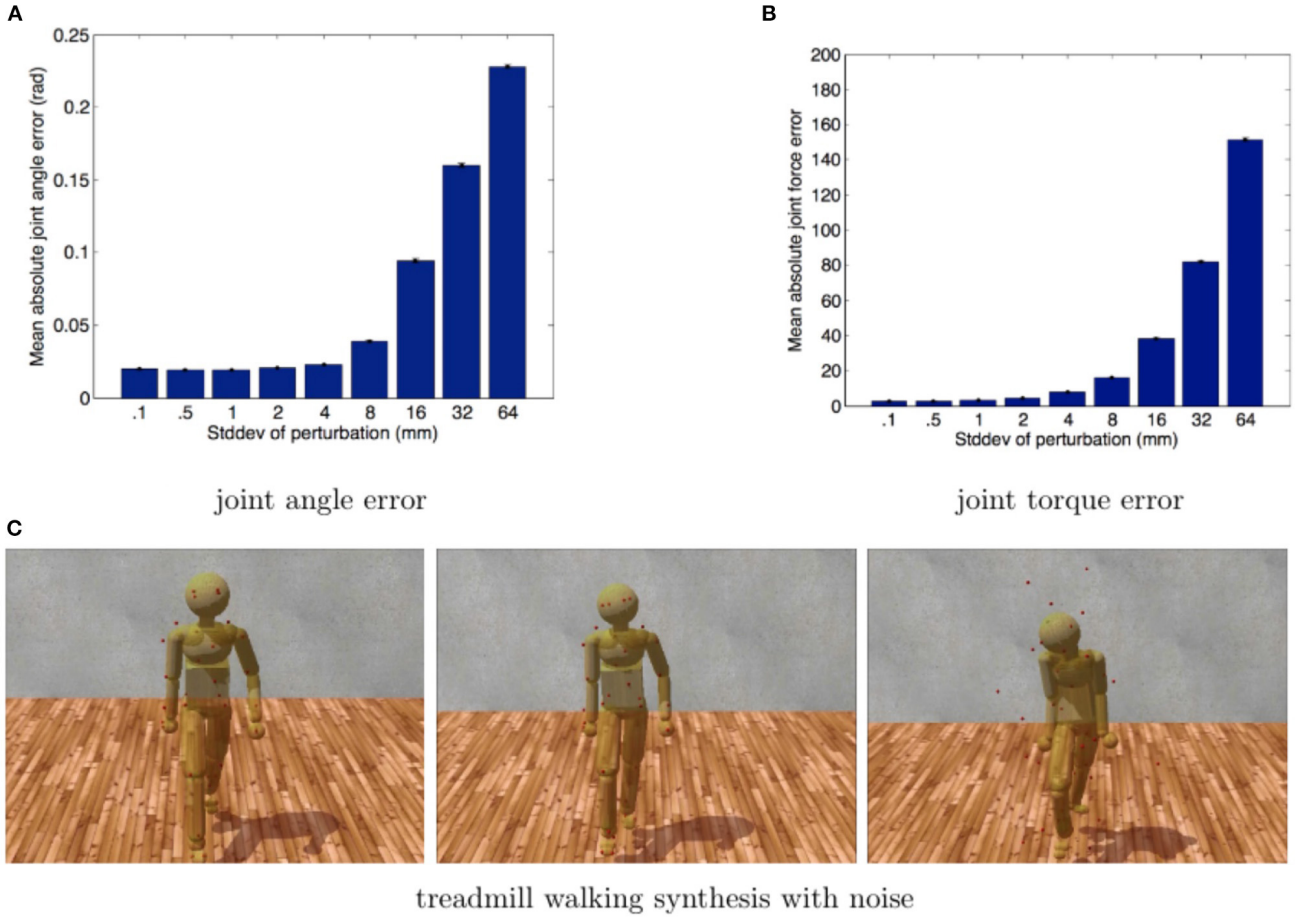
Model noise sensitivity. The errors of joint angles and internal torques, resulting from physics-based inverse kinematics and inverse dynamics, are used to analyze perturbed marker data. We repeated the process twenty times for each noise level at nine different standard deviations. Standard-deviations, in mm, were (0.1, 0.5, 1, 2, 4, 8, 16, 32, 64). Error bars show the standard error of the mean. **(A)** The accuracy of the PhaseSpace motion capture device is ~5 mm over its 3 × 6 m workspace, resulting in an average angular error of 1°. **(B)** The same estimates for torque error are between 5 and 10 Nm, typically ~1%. These small errors are well within the requirements for our experiments. **(C)** Poses generated by forward dynamics using forces obtained from three inverse dynamics simulations based on Gaussian perturbed walking data (0.1, 8, and 64 mm noise levels). Although at very high noise levels, the model follows the reference motion poorly, the movement still looks, qualitatively, like walking.

Using the synthetic marker data, we analyzed the process by perturbing all marker positions at each frame in time along all three axes with mean-centered Gaussian noise of a controlled standard deviation. Applying physics-based pose-fitting followed by inverse dynamics produced a new set of virtual marker positions, joint angles, and torques. The results are shown in Figure 6.

Gaussian perturbations render the marker data dynamically inconsistent. This dynamic inconsistency also pushes a constrained system toward singularity, making it more challenging to solve numerically. We included very high noise levels to see if they would slow the system down or prevent it from finding any solution. In all cases, the system analyzed the perturbed data and found poses to fit the marker data.

After running through an inverse kinematics pass, an inverse dynamics pass for each trial runs, we compared the marker attachment points, joint angles, and joint torques from the second pass to the synthetic ground truth data. Figure 6 shows the mean error across all degrees of freedom from eight steps walking. Although the perturbations make the marker data dynamically inconsistent, small amounts of noise have minimal effect on the computed measurements. Figures 6A,B standard error deviations show that functional effects are minimal up to 8 mm. A ±1 mm PhaseSpace marker position accuracy translates in our model into an average joint angle error of 0.02 radians and average force errors of 3 Newtons, which is acceptable for our experiments.

**Figure 7:**
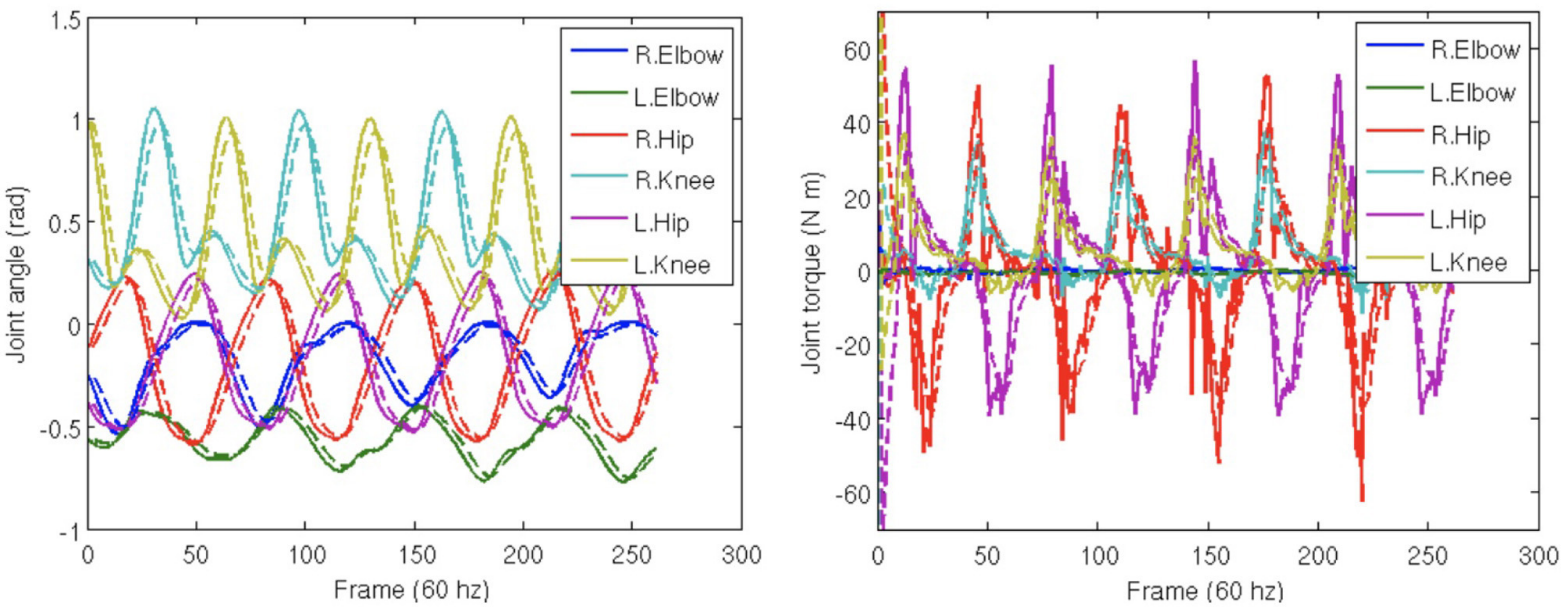
Trajectory reconstruction. Trajectories of selected degrees of freedom from the perturbation study. Solid lines show ground truth calculated from the motion capture data. Dashed lines show recovered data. Simulated spring forces make the computed data lag behind and smooth the ground truth.

There is a systematic error in both the marker positions and joint angles caused by the fact that the constraints behave like springs. The spring-like behavior causes the marker positions and joint angles to lag behind their targets by a small amount and dampens the overall movement. This lag and damping are apparent in Figure 7 comparing individual trajectories for selected dimensions of the joint angles and torques. As shown in Figure 7, the data follow ground truth very well under low noise conditions.

#### 3.1.2. Residual Torques/Forces and Ground Forces

In the classical inverse dynamic area, discrepancies between the model and humans that created the data necessitate non-realistic “residual” forces to keep the model from falling over when dynamically reproducing most movements. The HDM includes a joint to the model's waist to constrain it to reproduce orientation deviations found during the pose-fitting pass. To minimize the effect of these external forces, we used torque limits on the amount of stabilizing torque available.

**Figure 8:**
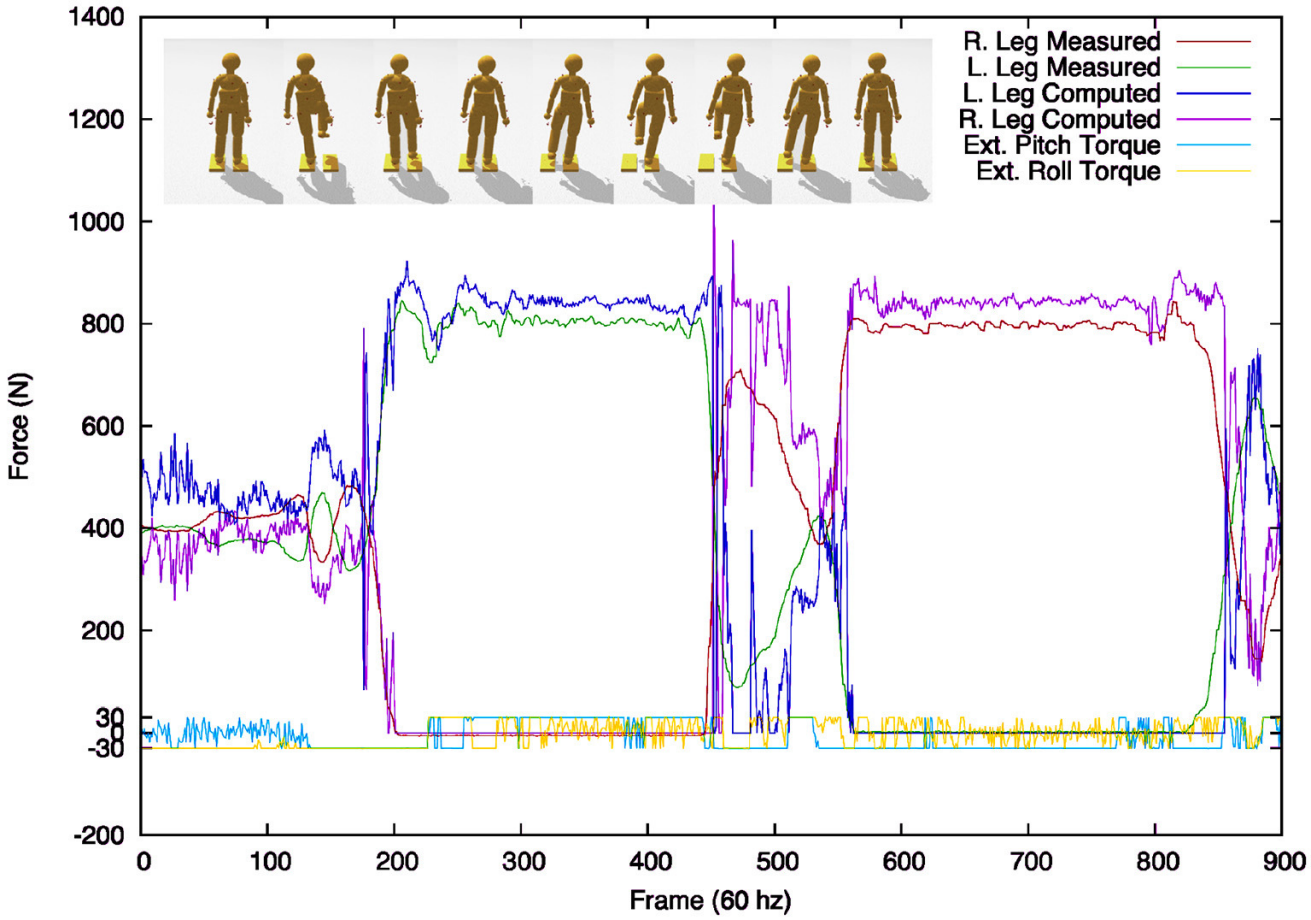
Comparing ground forces between the model and the Wii force plate. **(Top)** Two Wii force plates serve as accurate calibration reference. A subject stood on the two plates and then changed stances, balancing first on the left foot and next on the right. **(Bottom)** The comparison between the measurement systems is surprisingly good, during the stance phases. The 10% difference between the Wii measured ground forces and the *computed* forces can be reconciled by including the model's residual balance forces.

The fully configured system could be tested against an objective set of measurements. We compared HDM data together with ground force data from a pair of balance boards. Figure 8 shows the calibration of the ground force computed from our method compared to those taken from *Wii*^*TM*^ force plates. A subject standing on two force plates varied their stance from one being supported exclusively by leg standing on one plate and then shifted their weight to the other leg supported by the other plate. For this simple movement of transitioning from standing on one foot or the other, residual angular torques of 30 Nm were sufficient to keep the dynamic model quite close to its target trajectory.

The residual torques are very modest, being within ±5% of the maximum excursion. The correspondence is a little better as the faux vestibular balance forces are not factored into the comparison. Note also that we cannot expect the correspondence to be exact during the phase between the two stances as there is no attempt in the model in this test to make the dynamics of the changing stance match that of the force plates. To generate independent movements, such as grasping might need additional accuracy (Sentis et al., 2010), but for estimating a subject's energetic cost, the accuracy is well within range.

Figure 8 also shows the comparison results between the sensor-measured ground forces for the right and left feet (red and green lines) with the computed ground forces found through physics-based inverse dynamics (blue and pink lines). During the bipedal stance phase, the forces come surprisingly close. The most significant discrepancies come during the transition from one foot to the other. These discrepancies can be blamed mainly on poor collision detection resulting from an abstract model of the foot.

### 3.2. HDM Experimental Evaluation

The previous demonstrations report on tests of the fundamental performance of the system. This section focuses on three tests of the HDM's ability to fit experimental data. The first test uses a subject carrying out successively more difficult reaches in a virtual reality environment to test whether the model's estimate of movement costs correlates with increasing task difficulty. The second test simulates data from an issue facing movements in an aging population. Do aging subjects' reduced use of arm swing while walking incur a movement cost, and does the HDM's estimate correspond to laboratory treadmill data? The final test demonstrates an essential property of the model concerning its degrees of freedom. The critical observation is that virtues of their interconnections constrain the degree of freedom of the model; thus, the control of a posture can be achieved with a significantly reduced set of crucial marker positions. This property has implications for movement control programs.

#### 3.2.1. Experiment 1: Whole Body Reaching

The movement accuracy test is encouraging, but the importance of the method depends on its usefulness to capture the energetic cost of whole-body movements in a complex experimental setting. One such venue is a three-dimensional Virtual Reality (VR) environment. The advantage of the VR environment for studying human movements is that the dimensions and the dynamic variations of the parametric quantities describing the setting can be varied with full experimental control.

**Figure 9:**
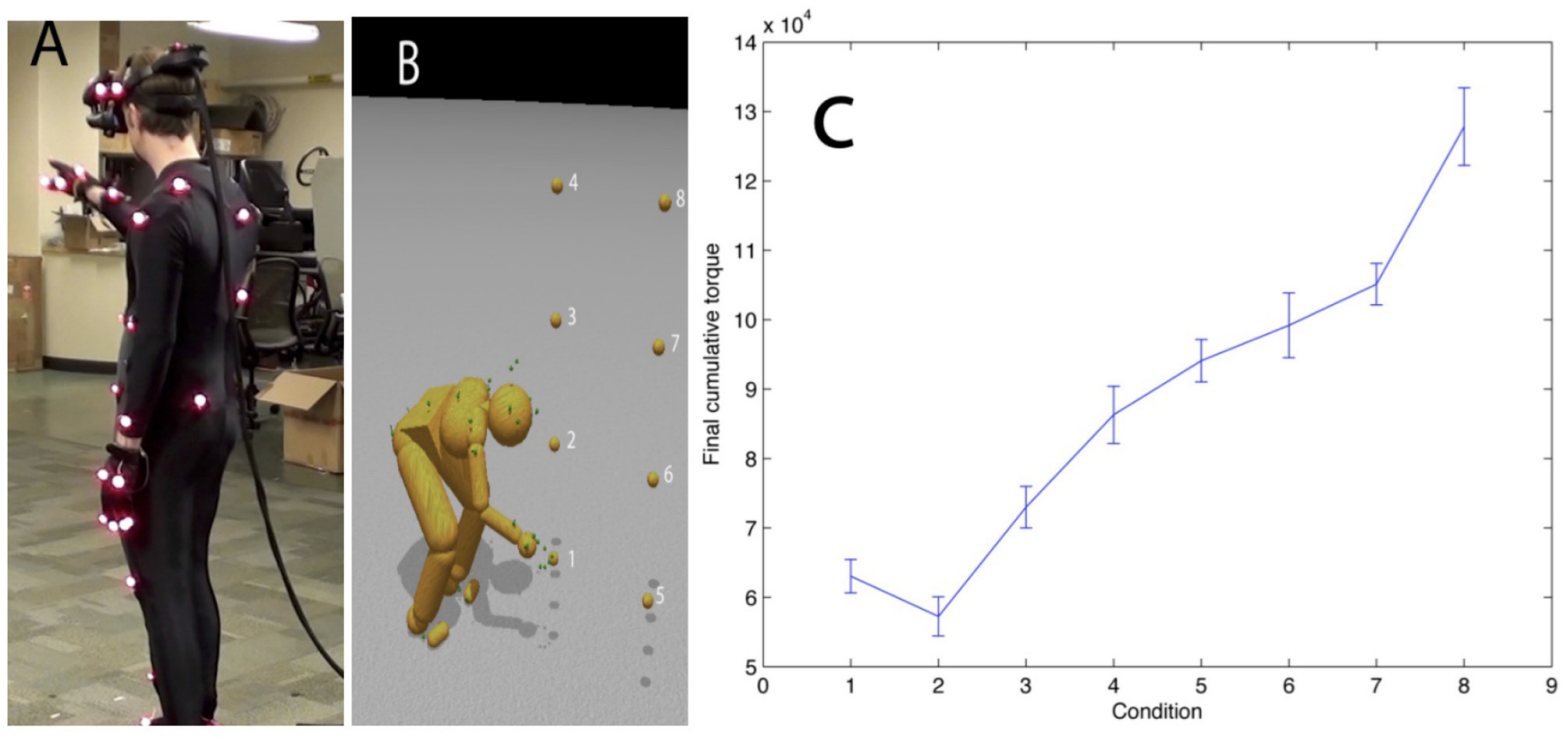
Reaching in a virtual reality environment. **(A)** A subject reaches to touch virtual targets seen in a HMD. The subject's reach is unconstrained. **(B)** The subject reaches to the different numbered targets on separate trials. **(C)** The average integrated torque over 10 trials per reach shows that the method reliably discriminates between movement costs for the further and higher locations.

In this experiment, we studied how a subject chose whole-body movements in reaching targets. Figure 9 demonstrates the experimental setup. The subject wears the PhaseSpace motion capture suit and the nVisor head-mounted stereo display. In each trail, the subject starts from a particular starting position marked with tape on the lab floor, approaches a target suspended in 3D space, and finally touches it.

Tests were able to establish that, just focusing on integrated net torque and avoiding stiffness, the relative cost of a movement recorded by our system reliably discriminates the energetic costs of the movement in the way hypothesized. The hypothesized cost of reaching for and touching each target was ranked based on distance and height relative to the subject. Note that target 2 is the least expensive as the subject does not have to crouch or extend significantly to touch it. Targets 5 through 8 are more costly than targets 1 through 4 as they require that the subject take a step to touch them. These results were expected, but the point was to show that the overall setting and model could produce reliable torque estimates.

This demonstration shows that the model can be used in any setting where the cost of a movement is hypothesized to be a constituent factor. We develop this technique further in the next demonstration.

#### 3.2.2. Experiment 2: The Cost of Stiff Arm Walking

Once the joint stiffness parameters were adjusted appropriately, can it reproduce the results of a stiffness modulating experiment? The experiment we tried was to replicate that of Ortega et al. (2008). They showed that arresting the arm swing during treadmill walking incurred an increased metabolic cost of 6%. Our hypothesis was that to reproduce this result we could modify our walking data for the model so that the arms were clamped by the sides with stiff stationary markers.

**Figure 10:**
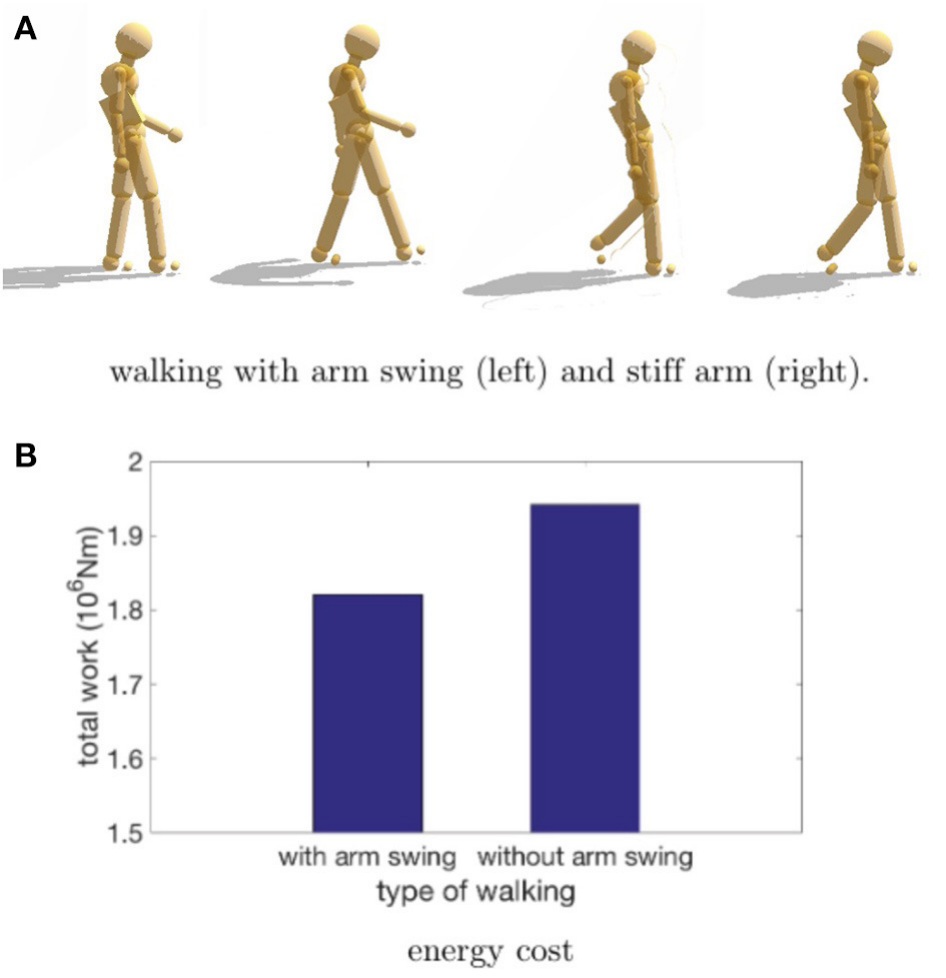
Comparison of efforts while walking with/without arm swing. **(A)** In a preliminary test of our design, the energetic cost of normal walking is compared to the case where the arms are constrained from swinging. Our hypothesis is that if subjects are instructed to walk without moving their arms, they will accomplish this by using muscle co-contraction and that this effect can be realized in the HDM with stationary markers that keep the arms vertical. **(B)** The increased cost measured by the HDM is 6.1%, extremely close to the 6% result obtained by Ortega et al. (2008).

To test this feasibility, we used one of our HDM walking data sets in a test situation. The cost of walking was computed with a modification designed to model the experimental protocol in Ortega et al. (2008). To simulate their experiment, we modified the model data so the arms could swing with the walking gait for the standard case, but for the restricted case, the arms were constrained by markers that move with the stride but are not allowed to swing. Since the arms under restricted situations could not balance the leg movements, we expected the energetic cost to be higher. As shown in Figure 10, the result was that the constrained walk was about 6% more expensive than the standard walk, which was essentially the value obtained by the Farley lab (Ortega et al., 2008). The use of the HDM in imitating this experiment shows off the model's utility; no elaborate tuning was necessary to obtain the preliminary result other than restraining the arms. It should be appreciated, that the model experiment required only hours to program, whereas the human experiment takes many days to set up, and the corresponding measure of cost is delicate.

#### 3.2.3. Experiment 3: Controlling Poses Using Reduced Marker Sets

Human pose sequences from simple single-behavior motions lie on a very low-dimensional linear subspace (Barbič et al., 2004). Previous research showed that for many movements, with suitable internal stiffness, it is only necessary to control the location of a reduced set consisting of the head, hands, and feet markers (Liu et al., 2006). This observation is the centerpiece of *uncontrolled manifold theory*, which restrict control to a subspace of the degrees of freedom, leaving the rest to the natural system dynamics (Scholz and Schöner, 1999; Torres-Oviedo and Ting, 2007). An obvious comes from ice skating. Pairs spinning on the ice would use one set of makers while jumping would use another set. Another example is using a subset of the markers to constrain the dynamics still produces reasonable walking gaits. This property could also have been expected from studies of muscle synergies, which show that muscle contractions coordinate in movement generation (Ting and Macpherson, 2005; Ting, 2007). As described above, the HDM uses 41 markers, which means a pose is represented by a 123-dimension coordinate system. Our test compares the generated pose using a reduced marker set with the one using the full marker set.Tests of movement accuracy revealed that the dynamics engine was able to function with significant fewer numbers of markers. Another benefit is that fewer markers can make interpolating across drop-outs in the data easier.

**Figure 11:**
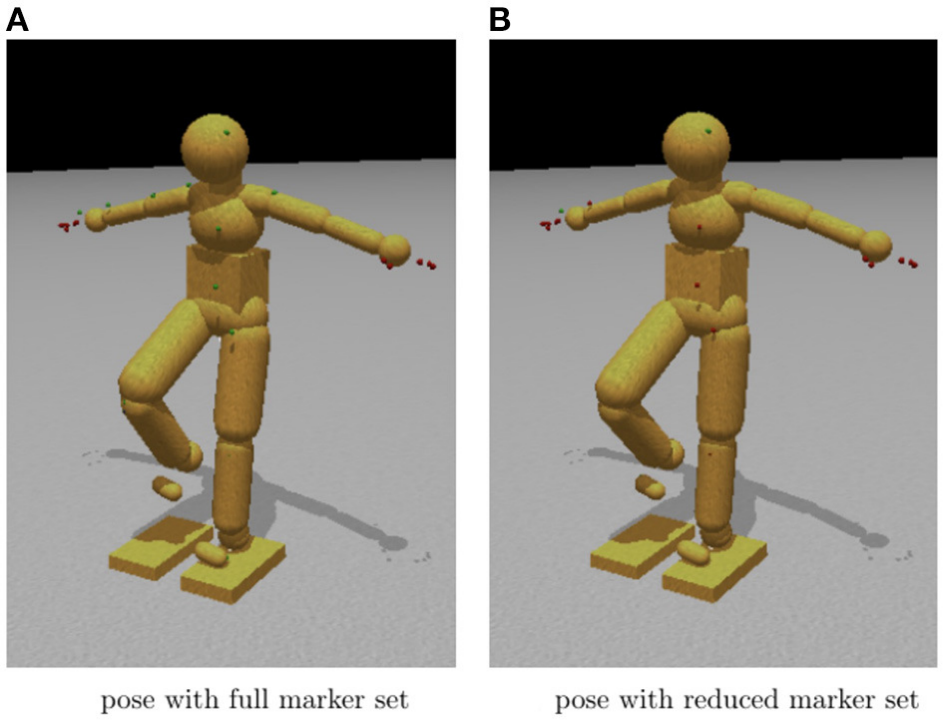
Movement control using dynamic synergies. **(A)** Body configuration using all marker constraints. Note the similarity to the sparsely constrained pose. **(B)** Body configuration using constraints on only the head, hands, and feet. In many cases, the pose found using a full set of marker constraints is quite close to that found by a sparse set of constraints. These two images show almost no differences between using a full or a sparse set of marker constraints.

Figure 11 shows a qualitative comparison between a pose found using the whole marker set (on the left) and one found using only head, hands, and feet (on the right). To achieve the reduced marker pose, we started the model in an upright stance with the arms by the side, and then the reduced set markers are moved slowly along trajectories that leave them in the final posture. The straight arms take advantage of the elbow joint angle limitation.

**Figure 12:**
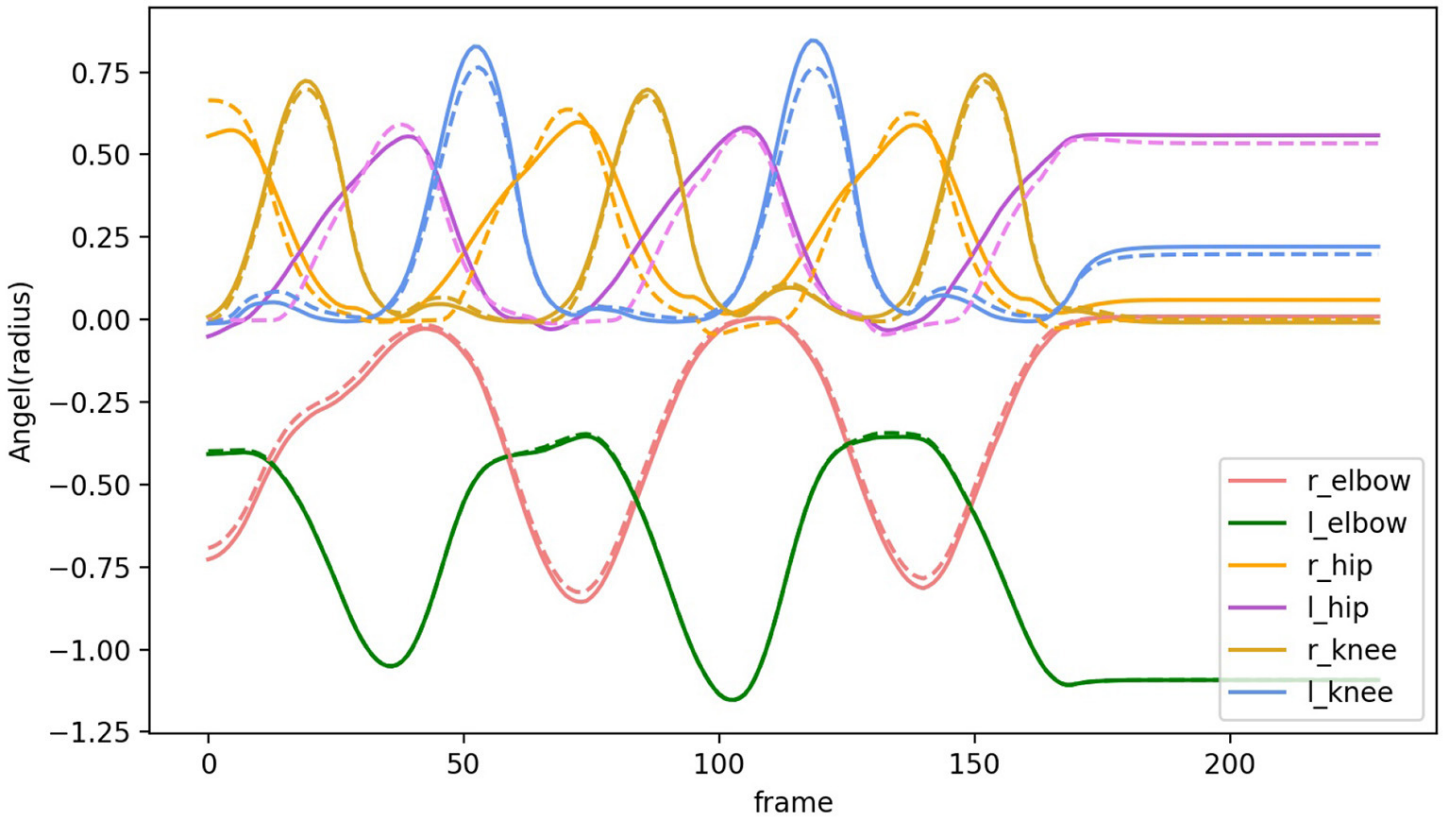
Comparison of joint angles along the selected degree of freedom. Solid lines show joint angles recovered based on the full marker set. Dashed lines show joint angels recovered based on a reduced marker set.

Joint limits on the knees and elbows and general joint stiffness naturally bias the physics engine to find a pose that is very close to the fully constrained pose. Body inertia and joint stiffness naturally clean up minor noise and occlusions in the captured marker data. The resulting joint angles in transit allow the specification of the complete set of dynamic torques. To test this feature of HDM quantitatively, the recovered joints angles while walking according to the reduced marker set were compared with those from the full marker set. Figure 12 illustrates the recovered joints angles are quite similar with the original joints angles.

This result has important general implications. First of all, the finding suggests that the kinematic plan for movements can be compressed into a subset of formative trajectories, leaving the remaining degrees of freedom interpolated using the body's dynamic constraint. Another aspect of this observation is that the reduced set can be used to adjust movements to individual circumstances, again leaving the detailed interpolation to the dynamics.

## 4. Conclusion and Discussion

This paper provides a method of building a humanoid model on top of a physical engine that can analyze and synthesize human movements. Its 48 degrees of freedom and generalized spring constraints allow models of scale that are robust to disturbances. In addition to being an analytical tool for experiments, it can also generate movements from a kinematic plan. The system has several features:

1. It utilizes the realizations of constraints as implicit springs. The spring parameters exhibit many advantageous properties. They stabilize the simulation, pushing a constrained system away from singularities, and reducing constraint error.
2. It calculates the movements' energetic cost to provide the capability to compare different movement scenarios. Achieving this goal can be tricky, owing to the lack of systems that can provide independent cost measures. The HDM achieved an excellent correspondence with force plates, as shown in the experiment to measure the human's stance change.
3. It produces correlations with similar tests with human subjects, such as our research with stiff-arm walking. Once we have vetted the system in many such areas, it can be used as a predictive tool, as in the experiment showing the different costs of reaching targets. We have developed a large-scale three-dimensional tracing experiment in virtual reality (Liu et al., 2019) to elicit natural whole-body movements under common goals. Our future work is to analyze the energetic cost using the HDM.
4. It shows that such a model can play a valuable role in studying the kinematic-plan model's consequences. In particular, the reduced degree of freedom control demonstration supports the *uncontrolled manifold* view wherein a subset of crucial degrees of freedom can direct a movement with the uncontrolled degrees of freedom interpolating the movement using the system's dynamics (Carpenter, 1968; Latash, 2008).

One way of illustrating the method's robustness is to combine a kinematic data set from the source with another set of dynamic parameters. So far, we have explored the HMD capability of using data from two other laboratories. One source is Carnegie Mellon University's graphics laboratory's motion capture database^10^. The other source is the motion data from the Hayhoe laboratory at the University of Texas psychology department, motion capture data from subjects traversing rough outdoor terrain. In tests, the data gathered with a different motion capture device is combined with the inertial data from another model to make a composite. We could use our dynamics calculation to compute joint torques for the hybrid system by adopting the imported database's marker conventions. Although the estimate is thus done for a synthetic pairing of kinematic data and dynamic parameters, this combination, the integration is stable and leads to identifiable torques.

Besides its use of a mechanism for interpreting experiments, the system can also serve as an adjunct for theorizing about the human system's organization concerning its space-time performance since many of these issues are open. While an enormous amount of research in human motor control has produced ever more refined subsystem components' elucidations, a comprehensive theory at the level of large-scale dynamics is still unsettled.

One question is a description of how the motor cortex can code information to drive the high temporal bandwidths of the spinal cord circuitry. Several possibilities were debated at the *Neural Control of Movement* conference in 2013 without definitive results. We have emphasized that the motor cortex communicates a coded kinematic plan together with stiffness settings that play an essential role in shaping the dynamics in muscle spindles (Blum et al., 2020).

Studies with kinematics coded with temporal basis functions have shown that a kinematic plan can be coded to reduce the bandwidth needed by a factor of ~10^3^ Hz (Iyer and Ballard, 2011; Won et al., 2020). More recently, sparse coding has been used to solve this problem (Glanz et al., 2021), despite unnecessary caution of its applicability to cortical motor areas (Beyeler et al., 2019). A tack that is still open would be our large sets of kinematic data to attempt to model cell responses in cortical area M1 to see if they turn out to be correlated with Graziano's data. The expectation would be that cells might have an informational interpretation analogous to the one used for oriented calls in the striate cortex. The facility with which the HDM system can collect motor data would allow a ready exploration of motor coding strategies similar to the visual domain. Being able to store movement segments would obviate the need to use an unlikely option to generate dynamic codes in the cortex neurally. This observation finds support in neural recordings that show movements generated cortically in discrete phases (Zimnik and Churchland, 2021), and the Boston Dynamics Atlas robot also uses a look-up strategy (Feng et al., 2014).

Regarding the uncontrolled manifold concept, a critical insight was the use of reduced degrees of freedom constraints in computing the dynamics. If the limitations are near the number of DOFs of the system, the torque recovery can quickly become numerically unstable. However, between 20 and 41 markers in the HDM provide sufficient constraints to integrate the dynamic equations reliably by allowing the system's natural dynamics to interpolate the motion appropriately.

In summary, the method has several advantages over alternative methods:

1. It can be easily implemented in a single robust framework of the physics engine. Using the physics engine for multiple tasks allows a unique human model to be used from start to finish, rather than being forced to use the conventions built into a commercial package.
2. The method is fast. The simulation engine is designed for performance, making it possible to analyze movement captured in real-time and create interactive experiments with stimuli dependent on the feedback results.
3. The software is free. Freely accessible code, such as ODE, is useful because it facilitates comparison and collaboration in research. Fourth, the method handles multiple ground contacts and noisy data challenging to related approaches. Kinematic loops do not require any special treatment. The method is robust even to large perturbations making data dynamically inconsistent.
4. The tunable parameters (CFM), couched in the physics framework, are intuitive. It is more straightforward to specify the importance of a constraint in force and mass rather than arbitrary gains and weightings. We illustrate these advantages by using ODE to analyze and reproduce movement recorded from optical motion capture.

There are several ways to improve the system in the future, and the following two are the most important among them. One limitation of our method for computing torque is that it is insensitive to muscle stiffness, which is both passive and can be actively modulated (Atkeson and Schaal, 1997; Awrejcewicz et al., 2012). Increasing stiffness will increase the overall net movement energetic cost and needs to be taken into account. The observation somewhat ameliorates this issue that subjects will try to minimize energetic costs in most natural tasks and thus exploit natural dynamics whenever they can, reducing high levels of co-contraction (Carpenter, 1968; Shadmehr and Arbib, 1992; Sternad and Sternad, 2009). However, the ubiquitous use of spring as a constraint means opening up the possibility of adding springs to the joint degrees of freedom to model stiffness. These could also have parametric programmable spring constants to model muscle co-contraction. The second feature that could be added is an improved system to keep the human model upright. Any of the three human sources of this needed information—visual, vestibular, and proprioception—would be candidates for this practical constraint. At present, the HDM uses a faux system of rotational torques at the center of gravity, but these could easily be replaced with more appropriate ankle torques.

## Author Contributions

JC created the initial protocol of the dynamic model and did some primary validations. LL further developed the model, conducted the experiments, and wrote the paper. DB designed the experiments and worked with LL on the overall paper editing and scientific presentation.

## Funding

This research was supported by the National Institutes of Health EY05729.

## Conflict of Interest

JC is employed by company Google Inc. The remaining authors declare that the research was conducted in the absence of any commercial or financial relationships that could be construed as a potential conflict of interest.

## Publisher's Note

All claims expressed in this article are solely those of the authors and do not necessarily represent those of their affiliated organizations, or those of the publisher, the editors and the reviewers. Any product that may be evaluated in this article, or claim that may be made by its manufacturer, is not guaranteed or endorsed by the publisher.

## Footnotes

^1^MuJoCo, http://www.mujoco.org/

^2^Bullet, https://pybullet.org/

^3^Havok, https://www.havok.com/

^4^OpenDE, http://www.ode.org/

^5^PhysX, https://developer.nvidia.com/gameworks-physx-overview

^6^HDM, https://github.com/EmbodiedCognition/HDM_UI

^7^HDM Demo, https://youtu.be/ASs4Wo5PQcM

^8^ODE CFM http://www.ode.org/ode-latest-userguide.html#sec_3_8_0

^9^HDM UI Demo, https://youtu.be/ASs4Wo5PQcM

^10^CMU Graphics Lab Motion Capture Database: http://mocap.cs.cmu.edu/
