# Supplementary material for: Computational Modeling: Human Dynamic Model
Source: Front Neurorobot. 2021 Sep 24;15:723428. doi: 10.3389/fnbot.2021.723428 (PMC8500180; doi:10.3389/fnbot.2021.723428)
Supplement: Supplementary file 1 [file Presentation_1.pdf]

Lijia Liu [1,*], Joseph L. Cooper [1,2] and Dana H. Ballard [1]

[1] *Department of Computer Science, The University of Texas at Austin, Austin, TX, USA*
[2] *Google Inc. Mountain View, CA, USA*

Correspondence*:
Lijia Liu
lijialiu@utexas.edu

SUPPLEMENTARY MATERIALS

S1 Appendix: Model mathematical derivation details

The principal insight in this section is that ODE can be used as an effective controller. We present a derivation of the mathematics underlying the physics simulation. The derivation comes from directly analyzing the ODE codebase, and it consequently differs from other derivations using Lagrange multipliers to arrive at the same final result (e.g., (Featherstone, 2014)). We present another derivation illustrating the equivalence between softened constraints in ODE and implicit springs.

When modeling human movements, we assume that the human body does not collide significantly with itself and so typically only process collisions between the model and the ground.

Notation

Physical simulation involves a large number of different variables to represent constraints and relevant physical quantities. Table 1 presents specific symbols and their meanings for reference.

Scalars are represented with lower-case, un-bolded symbols: x. Bold lower-case symbols represent column vectors, $\boldsymbol{x} = \begin{bmatrix} x_1 \\ x_2 \end{bmatrix}$. Bold, upper-case symbols to represent matrices. Dot-notation indicates time derivative: $\dot{x} = \frac{dx}{dt}$. The circumflex accent indicates a 3d vector being used as a skew-symmetric matrix representing a cross-product operation: $\hat{\boldsymbol{x}}\boldsymbol{y} = \boldsymbol{x} \times \boldsymbol{y}$

Coordinates are typically relative to a global reference frame. However, a tilde, $\tilde{\boldsymbol{x}}$, indicates a quantity that uses a local reference frame, e.g., a body-relative frame, rather than the global frame. We use subscripts to indicate that a quantity refers to a specific dimension, a particular rigid body, a point in time, but clarify the subscript's meaning when necessary to remove ambiguity. Table 1 introduces the primary symbols within the text.

For conciseness in notation, we typically combine angular and linear quantities as a single symbol. This representation is used both for position and orientation even though orientation does not conveniently fit into a 3×1 vector. Fortunately, angular velocity and angular acceleration, $\boldsymbol{\omega}$and $\dot{\boldsymbol{\omega}}$, do combine well with linear velocity and acceleration $\dot{\boldsymbol{x}}$and $\ddot{\boldsymbol{x}}$, and it is these quantities that feature primarily when dealing with a constrained system. We will also represent the state of multiple bodies using a single symbol when convenient. For example, for a system with two bodies, we will represent the combined linear and angular accelerations (a 12d vector) as $\ddot{\boldsymbol{x}}_t$. For this same 2-body system, Newton's law relating force, mass, and

Symbol	Meaning
$\boldsymbol{x}$	position or state of one or more rigid bodies
$\dot{\boldsymbol{x}}$	velocity (usu. linear and angular)
$\ddot{\boldsymbol{x}}$	acceleration (usu. linear and angular)
$\boldsymbol{R}$	rotation matrix representing orientation of a body
$\boldsymbol{\omega}$	angular velocity
$\boldsymbol{q}$	quaternion representation of an orientation or rotation
m	mass of a single rigid body
$\boldsymbol{M}$	mass matrix
$\boldsymbol{I}$	identity matrix
$\boldsymbol{\mathcal{I}}$	moment of inertia tensor
n_b, n_c	number of bodies, number of constraints
$\boldsymbol{\alpha}, \boldsymbol{\beta}$	stabilizing parameters added to the equations of motion
$\phi()$	error or energy function for a single constraint
$\boldsymbol{J}$	matrix of partial derivatives of constraint error functions
h	timestep
$\boldsymbol{f}$	forces (and torques)
$\boldsymbol{\tau}$	torques
$\boldsymbol{\lambda}$	constraint forces

Table 1. Meanings of specific symbols used to discuss dynamic simulation

acceleration is as follows:

$$\begin{bmatrix} \boldsymbol{f}_{1t} \\ \boldsymbol{\tau}_{1t} \\ \boldsymbol{f}_{2t} \\ \boldsymbol{\tau}_{2t} \end{bmatrix} = \begin{bmatrix} m_1\boldsymbol{I} & 0 & 0 & 0 \\ 0 & \boldsymbol{\mathcal{I}}_{1t} & 0 & 0 \\ 0 & 0 & m_2\boldsymbol{I} & 0 \\ 0 & 0 & 0 & \boldsymbol{\mathcal{I}}_{2t} \end{bmatrix} \begin{bmatrix} \ddot{\boldsymbol{x}}_{1t} \\ \dot{\boldsymbol{\omega}}_{1t} \\ \ddot{\boldsymbol{x}}_{2t} \\ \dot{\boldsymbol{\omega}}_{2t} \end{bmatrix} \Rightarrow \boldsymbol{f}_t = \boldsymbol{M}_t\ddot{\boldsymbol{x}}_t$$

where $\boldsymbol{I}$ and $\boldsymbol{\mathcal{I}}_{it}$ are 3×3 block matrices.

Dynamic State

Coordinates in the simulation world are defined relative to an arbitrary origin and basis set of directions. We refer to this inertial frame as the "global frame". Each rigid body also has its own point of reference and set of directions. Any point in the global frame can also be described relative to a body's frame of reference. It is convenient to define the point of reference of a body as its center of mass and use its principal inertial axes of symmetry as directions.

The position of the center of mass and orientation of a body within the global frame are here defined as $\boldsymbol{x}$ and $\boldsymbol{R}$ respectively. In 3d space, $\boldsymbol{x}$ is a 3×1 vector: $\boldsymbol{x}^{\boldsymbol{T}} = \begin{bmatrix} x, y, z \end{bmatrix}$ where x, y, and z are the distance from the origin along each of the three directions that establish the global frame of reference. For consistency, we deal with these distances in meters and assign "up" to the positive z axis.

Conservation of momentum makes it necessary to keep track of the time derivative of these quantities: $\dot{x}$ and $\dot{\boldsymbol{R}}$. Instead of explicitly representing $\dot{\boldsymbol{R}}$, it is convenient to keep track of the angular velocity: $\boldsymbol{\omega} = \begin{bmatrix} \omega_x, \omega_y, \omega_z \end{bmatrix}$ The relationship between these quantities is $\dot{\boldsymbol{R}} = \hat{\boldsymbol{\omega}}\boldsymbol{R}$ where $\hat{\boldsymbol{\omega}}$ is the skew-symmetric matrix commonly used to represent the vector cross-product So that $\hat{\boldsymbol{\omega}}\boldsymbol{x} = \boldsymbol{\omega} \times \boldsymbol{x}$.

Given n_b bodies, the dynamic state of the ith body at time t is its position, orientation, linear velocity, and angular velocity: $\left\{ \begin{array}{cccc} \boldsymbol{x}_{it} & \boldsymbol{R}_{it} & \dot{\boldsymbol{x}}_{it} & \boldsymbol{\omega}_{it} \end{array} \right\}$. We will assume that all of these values are framed in the global coordinate system unless specified otherwise. The body dynamics are also affected by the body's

constant mass m_i and inertia tensor $\boldsymbol{\mathcal{I}}_{it}$. The moment of inertia tensor, $\boldsymbol{\mathcal{I}}$, is indexed by time because the body's orientation changes how the the mass is distributed relative to the world frame: $\boldsymbol{\mathcal{I}}_{it} = \boldsymbol{R}_{it}\tilde{\boldsymbol{\mathcal{I}}}_i\boldsymbol{R}_{it}^{\mathrm{T}}$. We assume that the inertia tensor is constant relative to the body-local frame of reference (i.e., bodies are rigid).

In simulation, the forces $\boldsymbol{f}$ applied to the rigid bodies come from three general sources. These are constraint forces ($\boldsymbol{f_c}$), gravitational and gyroscopic forces ($\boldsymbol{f_g}$), and user/control forces ($\boldsymbol{f_u}$): $\boldsymbol{f} = \boldsymbol{f_c} + \boldsymbol{f_g} + \boldsymbol{f_u}$.

Integration Step

When a force is applied to a body, it translates into acceleration that is inversely proportional to the mass. Velocity is the time integral of acceleration, $\dot{\boldsymbol{x}}_t = \dot{\boldsymbol{x}}_{i0} + \int_0^t \boldsymbol{M}_i^{-1}\boldsymbol{f}_t dt$, and position is the time integral of velocity, $\boldsymbol{x}_{it} = \boldsymbol{x}_{i0} + \int_0^t \dot{\boldsymbol{x}}_{it} dt$. Because $\boldsymbol{f}_t$ may depend on $\boldsymbol{x}_t$ and $\dot{\boldsymbol{x}}_t$ as well as on discontinuous collisions and control inputs, analytic descriptions of body state are not usually possible. Instead we discretize the equations of motion and use a small, discrete timestep, h, to numerically approximate system dynamics. The most obvious thing to do is to linearize the force function, $\boldsymbol{f}_t$, and then take all the quantities from time t and use them to find the state at time $t + h$:

$$\dot{\boldsymbol{x}}_{t+h} = \dot{\boldsymbol{x}}_t + h\boldsymbol{M}^{-1}\boldsymbol{f}_t \tag{1}$$

$$\boldsymbol{x}_{t+h} = \boldsymbol{x}_t + h\dot{\boldsymbol{x}}_{t+h} \tag{2}$$

This "semi-implicit Euler" integration uses using the future velocity for computing position and is more stable than the standard formulation.

Although we lump orientation and position together as a single symbol, in practice there are a few distinctions that need mentioning. For example, gravity only applies to the linear state, while gyroscopic torques only apply to angular state. Gravitational forces are very straightforward, $\boldsymbol{f}_{\text{grav}} = \boldsymbol{M}\boldsymbol{g}$, where $\boldsymbol{g}$ indicates the direction and magnitude of gravitational acceleration.

Rotation is a non-linear phenomenon. However, we can approximate the motion of a rotating body by adding torques that imitate gyroscopic effects, see (Buss, 2000). Gyroscopic torques are applied to maintain conservation of angular momentum. Explicitly applying gyroscopic torques to bodies allows us to treat the rest of the system as though it conserved angular velocity rather than angular momentum. Thereafter, we can deal with the combined linear and angular quantities as a linear system.

The gyroscopic torques for each body are linearly approximated by

$$\boldsymbol{f}_{\text{gyro}} = \begin{bmatrix} 0 & 0 \\ 0 & \hat{\boldsymbol{\omega}}_t \end{bmatrix} \begin{bmatrix} 0 & 0 \\ 0 & \boldsymbol{\mathcal{I}}_t \end{bmatrix} \begin{bmatrix} 0 \\ \boldsymbol{\omega}_t \end{bmatrix}$$

These forces are zero if the three principal moments of the inertia tensor are equal. Otherwise, they represent the forces necessary for conservation of angular momentum. Unfortunately, this approximation tends to introduce energy into the system. We have reduced this problem in ODE by adding in additional terms as described in (Buss, 2000).

The constrained system is solved using mostly accelerations and velocities. At the end, however, it is necessary to integrate the velocities into new positions and orientations. Position and orientation are updated differently. For position, it is sufficient to multiply the linear velocity by the timestep and add it to

the current position. Adding angular velocity to orientation is not as straightforward. We integrate angular velocity into orientation by converting $\boldsymbol{\omega}_{i(t+h)}$ into a quaternion and then use the quaternion to rotate the current orientation forward in time following (Grassia, 1998).

Constraint Equation

When a rigid body is moving or spinning freely through space, the integration equations are sufficient to simulate dynamics. Adding constraints modifies the bodies' movements. Maintaining a relationship between two bodies requires forming a constraint on the state of the bodies. The integration equations tell us how to go from force to velocity and from there to position and orientation. To simulate an articulated model using maximal coordinates, we need to know what forces constraints apply to the bodies in the system.

To find the constraint forces, one must be able to mathematically describe the constraint. We define a multi-dimensional function over the combined position and orientation of all bodies in the system, $\boldsymbol{\phi}(\boldsymbol{x}_t)$, that produces a vector of size n_c specifying how much each constraint is violated, where n_c is the number of constraints acting on the system. For example, if the ith constraint keeps body b_2 a distance d above body b_1 in the z direction, we would have $\phi_i(\boldsymbol{x}) = x_{2z} - x_{1z} - d$. If b_2 is not separated from b_1 by a distance of d in the z direction, $\phi_i(\boldsymbol{x})$ reports the signed magnitude of that constraint error. For additional information on forming constraint equations, see (Featherstone, 2014; Smith, 2005).

In general, the error for a constraint is non-zero. Given a measure of the error for a given state, we seek to find constraint forces, $\boldsymbol{f_c}$, that reduce the error over subsequent time steps(Baumgarte, 1972). Specifically, over the timestep h, we seek a force to reduce the magnitude of the constraint error by a fraction α. That is

$$\boldsymbol{\phi}(\boldsymbol{x}_{t+h}) = (I - \boldsymbol{\alpha})\boldsymbol{\phi}(\boldsymbol{x}_t) \tag{3}$$

where $\boldsymbol{\alpha}$ is a $k \times k$ diagonal matrix with each $\alpha_i \in [0, 1]$ representing the fraction of error reduction over a time step. In ODE, the α value is controlled through the *error reduction parameter* (ERP) which can be set independently for each constrained degree of freedom. In practice, it is not possible to remove constraint error completely $(\alpha = 1)$ when using maximal coordinates because of error introduced by the various approximations employed to make the simulation linear and fast. Values of α typically fall within $[0.2, 0.8]$. Manipulating this value results in useful elastic and damping effects discussed later.

We use the symbol $\boldsymbol{J}_t$ to represent the $n_c \times 6n_b$ matrix of partial derivatives of $\boldsymbol{\phi}(\boldsymbol{x}_t)$. This matrix is a linear approximation of how the constraint error for each of the n_c constraints changes when the positions and orientations of the bodies change. Finding the constraint forces that satisfy Eq. 3 involves removing all references to unknown future quantities. The Taylor expansion of $\boldsymbol{\phi}(\boldsymbol{x}_{t+h})$ at $\boldsymbol{x}_t$, truncated after the first order term, approximates the future constraint error:

$$(I - \boldsymbol{\alpha})\boldsymbol{\phi}(\boldsymbol{x}_t) = \boldsymbol{\phi}(\boldsymbol{x}_{t+h}) \approx \boldsymbol{\phi}(\boldsymbol{x}_t) + \boldsymbol{J}_t(\boldsymbol{x}_{t+h} - \boldsymbol{x}_t) \tag{4}$$

This truncation has the effect of treating all constraints as linear. Many constraints used to simulate various joints are linear; others, however, contain higher-order terms and this truncation is one potential source of error in simulation.

Combining the two integrator equations, Eqs. 1 and 2, gives the future position/orientation in terms of the present position, velocity, and forces:

$$\boldsymbol{x}_{t+h} = \boldsymbol{x}_t + h\dot{\boldsymbol{x}}_t + h^2\boldsymbol{M}_t^{-1}\left(\boldsymbol{f}_{ct} + \boldsymbol{f}_{gt} + \boldsymbol{f}_{ut}\right) \tag{5}$$

Equations 3, 4, and 5 combine to eliminate all references to future quantities:

$$(I - \boldsymbol{\alpha})\boldsymbol{\phi}(\boldsymbol{x}_t) = \boldsymbol{\phi}(\boldsymbol{x}_t) + \boldsymbol{J}_t\left(\boldsymbol{x}_t + h\dot{\boldsymbol{x}}_t + h^2\boldsymbol{M}_t^{-1}\left(\boldsymbol{f}_{ct} + \boldsymbol{f}_{gt} + \boldsymbol{f}_{ut}\right) - \boldsymbol{x}_t\right) \tag{6}$$

This leaves one unknown vector at time t: the constraint forces $\boldsymbol{f}_{ct}$. Rearranging and simplifying, we get

$$\boldsymbol{J}_t\boldsymbol{M}_t^{-1}\boldsymbol{f}_{ct} = -\frac{1}{h^2}\boldsymbol{\alpha}\boldsymbol{\phi}(\boldsymbol{x}_t) - \frac{1}{h}\boldsymbol{J}_t\dot{\boldsymbol{x}}_t - \boldsymbol{J}_t\boldsymbol{M}_t^{-1}\left(\boldsymbol{f}_{gt} + \boldsymbol{f}_{ut}\right) \tag{7}$$

Note that in rearranging the terms this way, we divided both sides by the squared timestep, h^2, effectively changing the problem from one dealing with positions to one dealing with accelerations. This conversion is possible because of the relationship established between acceleration and position by the semi-implicit Euler integrator.

Equation 7 is almost the equation that ODE solves when simulating physics. The right hand side is a desired acceleration. The first term on the right is the acceleration that would result in a velocity that would remove a fraction (α) of the constraint error. The second and third terms account for the effects of momentum (current velocity), gravity, and other forces (e.g., user control forces) applied to the bodies. Each constraint becomes its own dimension in a "constraint space". The Jacobian matrix $\boldsymbol{J}$ projects accelerations from global forces into constraint space.

In general, the matrix on the left hand side of Eq. 7 is not square, making the problem under-constrained (or in some cases, potentially over-constrained). However, we can use d'Alembert's principle(Lanczos, 2020) to restrict the constraint forces to lie in the constraint space.

Another method for arriving at the constraint equation is through the use of Lagrange multipliers. Consequently, the constraint-space forces are typically denoted with λ. The Jacobian transpose gives the relationship between a force applied in constraint space and force/torque applied in the full coordinate space: $\boldsymbol{f}_{ct} = \boldsymbol{J}_t^{\mathrm{T}}\boldsymbol{\lambda}_t$.

The vector, $\boldsymbol{\lambda}_t$, holds the generalized forces applied by each constraint on all the bodies involved in that constraint, whereas $\boldsymbol{f}_{ct}$ holds the sum of the constraint forces applied to each individual degree of freedom of each rigid body. The LHS of Eq. 7 can then be rewritten as $\boldsymbol{J}_t\boldsymbol{M}_t^{-1}\boldsymbol{J}_t^{\mathrm{T}}\boldsymbol{\lambda}_t$, where $\boldsymbol{J}_t\boldsymbol{M}_t^{-1}\boldsymbol{J}_t^{\mathrm{T}}$ is now a $n_c \times n_c$ positive semi-definite matrix.

Returning to maximal coordinates, we will compress Eq. 7 down to

$$\boldsymbol{J}\boldsymbol{M}^{-1}\boldsymbol{J}^{\mathrm{T}}\boldsymbol{\lambda} = \boldsymbol{w} \tag{8}$$

In general, the matrix $\boldsymbol{J}\boldsymbol{M}^{-1}\boldsymbol{J}^{\mathrm{T}}$ may be singular. It is very easy to end up with redundant or conflicting constraints. For example, a box resting on the ground may get a contact constraint at each corner. If each contact prevents interpenetration and sliding (i.e., applies friction) then the contacts constrain a total of 12 degrees of freedom on a single rigid body with only 6 degrees of freedom to be constrained. Conflicting or redundant constraints can break the solver if not dealt with beforehand. The means for dealing with the conflict is clever. The physics engine softens the constraint, allowing it to "slip" proportional to the amount of force necessary to maintain it.

Because mass is always positive, the force, λ, applied to a particular constraint and the resulting constraint-space acceleration will have the same sign. Softening the constraint is therefore a matter of subtracting a scaled copy of λ from the desired acceleration (the right hand side): $\boldsymbol{J}\boldsymbol{M}^{-1}\boldsymbol{J}^{\mathrm{T}}\boldsymbol{\lambda} = \boldsymbol{w} - \boldsymbol{\beta}\boldsymbol{\lambda}$, where $\boldsymbol{\beta}$ is an $n_c \times n_c$ diagonal matrix of (typically small) non-negative values. This subtraction, of course, is equivalent to adding $\boldsymbol{\beta}$ to the LHS. Adding these small values to the diagonal of the effective inverse-mass-matrix makes the constraints seem lighter to the solver and moves the matrix away from singularity:

$$\left(\boldsymbol{J}_t\boldsymbol{M}_t^{-1}\boldsymbol{J}_t^{\mathrm{T}} + \boldsymbol{\beta}\right)\boldsymbol{\lambda}_t = -\frac{1}{h^2}\boldsymbol{\alpha}\boldsymbol{\phi}(\boldsymbol{x}_t) - \frac{1}{h}\boldsymbol{J}_t\dot{\boldsymbol{x}}_t + \boldsymbol{J}_t\boldsymbol{M}_t^{-1}\left(\boldsymbol{f}_{gt} + \boldsymbol{f}_{ut}\right) \tag{9}$$

The original programmers built soft constraints into the ODE simulation code. The variable, β, tunable for each constraint, is known in ODE as the *constraint force mixing* parameter (CFM). At first glance, the addition of these parameters may seem loose and unprincipled. However, correctly setting the parameters, α and β, changes a hard constraint into a simulated implicit spring with first order integration (see (Smith et al., 2005)).

It is well-known that the formula for ideal damped spring force is identical to the formula for PD control. However, connecting these two facts, namely that (1) ODE's constraints are mathematically equivalent to implicit damped springs and (2) damped springs are equivalent to PD controllers, has not been exploited. This insight is key to the success of the methods presented here. Our derivation shows that ODE's constraints are, in fact, stable PD controllers along with examples of how to take advantage of this fact. We proceed by discussing proportional-derivative control and the mass-spring-damper equation.

Implicit Simulated Springs

Proportional-derivative (PD) control is a common method used to compute forces that drive a system toward a target state. The PD control equation is the same as a mass-spring-damper system. There are two parameters, k_p and k_d. The control force f_{ut} at any instant in time is a function of the current position and velocity of the effective mass being controlled relative to its target:

$$f_{ut} = -k_p x_t - k_d \dot{x}_t \tag{10}$$

Discrete sampling of these forces, however, ruins the stability conditions. The potential for instability is apparent if we consider a mass m that only experiences damping forces. Using the semi-implicit Euler integrator, Eq. 1, we plug in the damping forces from Eq. 10 to get

$$\dot{x}_{t+h} = \dot{x}_t - \frac{hk_d}{m}\dot{x}_t = \left(1 - \frac{hk_d}{m}\right)\dot{x}_t \tag{11}$$

Time (t), mass (m), and damping (k_d) should all be non-negative values. It is clear, then, from this equation, that if $\frac{hk_d}{m} > 2$, the velocity will oscillate between positive and negative values and grow in magnitude. This oscillation rapidly causes the simulation to "explode" and is annoyingly common when using PD control. Overly stiff springs hit a similar limit with explicit discrete integration that causes them to gain energy and explode. Consequently, explicit PD control gains are tricky to tune. They must fall within certain limits that depend on the timestep and the effective mass experienced by the system.

The reason for this instability lies in the discrete integration, which is similar to approximating the area under a curve as the sum of multiple rectangles computed forward from the present. One solution is to

solve for the forces *implicitly*. Implicit integration is similar to approximating the area under a curve with fixed-width rectangles that end rather than begin on the curve. Rather than overestimate, this method tends to underestimate the area under an exponential curve. The resulting system does not explode, although it tends to dissipate rather than conserve energy. The implicit form of the damped-spring law depends on the integrator it is applied to. Being 'implicit', in this case, specifies that spring forces are computed from the *future* state of the system. Consequently, Eq. 10 becomes the following, (note the changed temporal indices):

$$f_{ut} = -k_p x_{t+h} - k_d \dot{x}_{t+h} \tag{12}$$

We do not know the future position or velocity, but using the integrator equations, Eqs. 2 and 1, we reframe Eq. 12 in terms of the current quantities and then solve for f_{ut} to get

$$f_{ut} = -\frac{k_d \dot{x}_t + k_p x_t + h k_p \dot{x}_t}{1 + m^{-1} h k_d + m^{-1} h^2 k_p} \tag{13}$$

If we analyze a pure damped system as before but using Eq. 13, we end up with

$$\dot{x}_{t+h} = \dot{x}_t - \frac{h k_d \dot{x}_t}{m + h k_d} = \frac{m}{m + h k_d} \dot{x}_t$$

The consequence of this relationship is that every constraint in ODE can be thought of as an implicit spring. An essential feature of this formulation is that the equations are solved simultaneously. When the implicit springs are solved simultaneously in the physics framework, the forces account for each other; the system would be fragile without this change. Softening the constraints to springs makes it so that we can solve a system that would otherwise be over-constrained. We can add more constraints than there are degrees of freedom.

Solving with Complementarity Conditions

For simplicity, we compress Eq. 9 down to $\boldsymbol{A}\boldsymbol{\lambda} = \boldsymbol{w}$. When $\boldsymbol{A}$ is non-singular, we can solve for $\boldsymbol{\lambda}$ by inverting, or preferentially, using a fast, numerically-stable solver such as a Cholesky decomposition. Some constraints, however, come with additional conditions that need to be solved with extra machinery. In simulation literature, these are known as inequality constraints. For example, a contact constraint keeps two bodies from moving towards each other by defining an error function: the separation of the contacting surfaces in the direction of one of the surface normals. If the surfaces overlap, then the error function has a negative value, and a positive constraint force will accelerate the surfaces apart. This acceleration is as it should be. However, the linear system also applies forces to correct a positive error, so the same constraint would also prevent the surfaces from separating.

The solution to this problem is to limit the amount of force available for satisfying the constraint. A contact constraint, in particular, limits the force to be non-negative. Contact friction constraints are limited on both sides to be proportional to the normal contact force. This limitation places upper and lower bounds on the constraint force variable: $\boldsymbol{\lambda}_{\text{lo}} \leq \boldsymbol{\lambda} \leq \boldsymbol{\lambda}_{\text{hi}}$, allowing constrained bodies to accelerate without bounds if the force necessary to hit the acceleration target falls outside of the limits. In ODE, the result is three possible conditions to satisfy a constraint:

$$\boldsymbol{a}_i\boldsymbol{\lambda} = w_i \text{ with } \lambda_i \in [\lambda_{i\text{lo}}, \lambda_{i\text{hi}}], \quad \boldsymbol{a}_i\boldsymbol{\lambda} > w_i \text{ with } \lambda_i = \lambda_{i\text{lo}}, \quad \boldsymbol{a}_i\boldsymbol{\lambda} < w_i \text{ with } \lambda_i = \lambda_{i\text{hi}}$$

where $-\infty \leq \lambda_{i\text{lo}} \leq 0 \leq \lambda_{i\text{hi}} \leq \infty$.

A linear solver cannot handle these extra conditions on the constraint forces. To solve this type of system, physics engines employ a mixed Linear Complementarity Problem (mLCP) solver. ODE offers two different solving methods for satisfying constraints under limited-force conditions. One method, known as Projected-Gauss-Seidel, solves constraints iteratively and accumulates the effects (Catto, 2005). Iterative methods tend to be faster and inaccurate when the system is near-singular or ill-conditioned. Simulated humanoid systems, particularly with two feet on the ground, tend to behave badly with this faster solver. The slower, pivot-based method follows the algorithm presented by Baraff (Baraff, 1994). Baraff's method is still easily fast enough for our purposes.

Each row in matrix $\boldsymbol{A}$ represents a constraint. The corresponding values of $\boldsymbol{w}$ and $\boldsymbol{\lambda}$ represent a "target" acceleration along the degree of freedom constrained by that row and the generalized force used to achieve it. For the ith row of $\boldsymbol{A}$, the diagonal element, a_{ii}, behaves like the inverse mass of the constraint. A force, λ_i, imposes an acceleration of $a_{ii}\lambda_i = w_i$ within the constraint error-space. The rest of the elements in a row of $\boldsymbol{A}$ encode the force's effects on other constraint dimensions. A change in the ith constraint force λ_i affects the jth constraint space by accelerating it according to $\delta w_j = a_{ij}\delta\lambda_i$. The order of the constraints is arbitrary, and they can be rearranged as long as every row-swap is accompanied by the corresponding column-swap that maintains the proper symmetry.

Baraff's solving algorithm (based on Dantzig's simplex method) takes advantage of this arbitrary ordering by dividing constraints into different sets: a satisfied set S, a limited set N, and an unaddressed set U. All constraints fit into one of these categories. The first step in finding a solution is to reorder and satisfy all the unlimited constraints without considering the rest, using a basic linear solver. The resulting system looks like

$$\begin{bmatrix} \boldsymbol{A}_{11} & \boldsymbol{A}_{12} \\ \boldsymbol{A}_{12}^{\text{T}} & \boldsymbol{A}_{22} \end{bmatrix} \begin{bmatrix} \boldsymbol{\lambda}_1 \\ \boldsymbol{0} \end{bmatrix} = \begin{bmatrix} \boldsymbol{w}_1 \\ \boldsymbol{A}_{12}^{\text{T}}\boldsymbol{\lambda}_1 \end{bmatrix} \tag{14}$$

Set S holds the rows of $\boldsymbol{A}_{1i}$. Set U holds the rest. Each constraint's target conditions can be represented as a piecewise line through force-acceleration space. We will call this multi-segmented line the target manifold for each constraint. Viewing constraints this way is another contribution of this work. The diagonal element of $\boldsymbol{A}$ associated with the constraint gives the slope of a line through the origin that represents the relationship between force (λ) and actual acceleration (A_{ii} is the effective inverse-mass of the ith constraint). The solver seeks to find a joint solution so that, for all rows of $\boldsymbol{A}$, the pairs of (λ_i, w_i) fall on the acceptable manifold. Forces from other constraints move the entire manifold up or down relative to the origin.

The β parameter takes the horizontal portion of the target manifold and tilts it so that when bigger forces are used, there is a lower target acceleration. Hence the constraint is spring-like. The vertical portions of the constraint represent places where the constraint has hit its force limits. That constraint can apply no additional force, so the acceleration must be allowed to increase freely. Otherwise, the constraint would be "obligated" to apply more force to get closer to its target acceleration.

Constraints are addressed one at a time. When dealing with ground contact force without softened constraints, once the solver found a sufficient force to keep a body from penetrating the ground, any remaining ground contact constraints would have nothing to do, resulting in an inappropriate distribution

of ground forces. With spring-like constraints, if one contact constraint supporting a body reaches its target force/acceleration, a second, redundant contact constraint will see whatever distance remains between the current acceleration and the target. Forces applied by the second constraint attempting to reach its target push the target manifold of the first constraint toward the origin. The force required to achieve the first constraint's target decreases until the forces balance appropriately. The balancing forces make it possible to more accurately compute inverse dynamics forces.

The algorithm for solving the mLCP progresses through each unaddressed constraint, one at a time, and finds the change in forces that will satisfy the new constraint without moving any of the current constraints off their piecewise target. Each iteration of the algorithm draws a new constraint from the unaddressed set U and addresses the change in force, λ, that will satisfy the new row without pushing any previously addressed rows off their manifold until the new row can be added to S or N. In the process, other rows may change between sets S and N, but each row remains on its target manifold in acceleration/force space. Consider this partitioned matrix:

$$\begin{bmatrix} \boldsymbol{A}_{11} & \boldsymbol{A}_{12} & \boldsymbol{a}_{13} \\ \boldsymbol{A}_{12}^{\mathrm{T}} & \boldsymbol{A}_{22} & \boldsymbol{a}_{23} \\ \boldsymbol{a}_{13}^{\mathrm{T}} & \boldsymbol{a}_{23} & a_{33} \end{bmatrix} \begin{bmatrix} \boldsymbol{\lambda}_1 \\ \boldsymbol{\lambda}_2 \\ 0 \end{bmatrix} = \begin{bmatrix} \boldsymbol{w}_1 \\ \boldsymbol{v}_2 \\ v_3 \end{bmatrix} \tag{15}$$

Adding a new force, λ_3, will change the accelerations of the other constraints. Accelerations of constraints at their limit are allowed to change, but those in set S must remain at their target. So we find the $\delta\lambda_3$ that moves v_3 toward w_3 and find the simultaneous $\delta\boldsymbol{\lambda}_1$ that keeps constraints in S satisfied. The constraint force takes the largest step that will not push any row out of its setting. This step will either satisfy the constraint or move another constraint to an intersection point on its manifold. We pivot the sets around and continue until all of our rows are in S or N. For additional detail, see (Baraff, 1994).

Recognizing that the solver deals with each constraint target as a piece-wise linear manifold provides valuable insight into how the simulation mechanism can be improved. We can make spring constraints that get more or less stiff as additional force is required. We can also introduce constraints with "deadzones" in their PD control. This type of constraint allows us to introduce controllers that only come into play when a dimension of interest drifts out of an acceptable range. This type of controller takes inspiration from the idea of "uncontrolled manifolds" in human motor control theory (Scholz and Schöner, 1999). With this constraint acting as a controller, the controller does nothing if a perturbation will not hurt performance.

From deadzone controllers, we can introduce novel constraints with secondary targets. A constraint whose forces and accelerations fall within acceptable tolerances has the flexibility to "help" another constraint that has reached its limit. For example, we can specify a target range for the knee, hip, and ankle joints of a simulated character. When these leg joints fall within their stated ranges, they can be allowed to pursue a secondary goal, such as keeping the torso upright or at a given height. This type of constraint can serve as a method for reducing the need for unrealistic residual forces. Removing residual forces implies deviating from original kinematic data. Constraints with secondary targets make it intuitive and clear how this deviation will occur can be extremely beneficial when using the simulation engine for analyzing and synthesizing movement data. We have created and submitted code for allowing controller constraints with a deadzone in acceleration space. [1]

[1] Full implementation of secondary targets for constraints is still in progress. It promises to help create intelligent constraint-based controllers.

S2 Appendix: Model dimensions determination based on marker data

The model consists of n_b rigid bodies connected by n_j joints. In this case, each joint consists of three to five constraints. Each joint connects two rigid bodies with anchor points (center of rotation) defined in the reference frame of both bodies. The joint constraints keep the anchor points relative to the two bodies together in the global frame. If bodies b_j and b_k are connected, a joint constrains them together at a common point. The joint anchor relative to body b_j is $\tilde{\boldsymbol{c}}_{jk}$. The anchor for body b_k is $\tilde{\boldsymbol{c}}_{kj}$. The joint constraint drives these points together in the global frame, creating three constraint rows:

$$\boldsymbol{\phi}_{jk} = \boldsymbol{R}_j \tilde{\boldsymbol{c}}_{jk} + \boldsymbol{x}_j - \boldsymbol{R}_k \tilde{\boldsymbol{c}}_{kj} + \boldsymbol{x}_k \tag{16}$$

The locations of these anchor points determine the segment dimensions (bone lengths) of the character model. Markers, each assigned to a specific rigid segment, represent a point on the human body. We seek anchor points that allow markers to remain approximately stationary relative to their assigned body segment. It is generally impossible to precisely find such a configuration (without creating an unreasonable number of body segments) because of soft-tissue artifacts (STAs). Skin and joints are not rigid. They stretch and give as muscles pull the bones. Modeling the body in maximal coordinates provides a way to model STAs explicitly.

Given a pre-defined model topology and markers assigned to specific model segments, we seek to find the joint anchor points between segments and the marker attachment points relative to the model segments. If the ith marker is assigned to the jth rigid body ($\boldsymbol{p}_i \to b_j$) at relative point $\tilde{\boldsymbol{s}}_{ij}$, we model the marker's attachment as a three dof constraint: $\boldsymbol{\phi}_{ij} = \boldsymbol{p}_i - \boldsymbol{R}_j \tilde{\boldsymbol{s}}_{ij} - \boldsymbol{x}_j$

The process models markers from an arbitrary point in time as infinite point masses. As bodies of infinite mass, constraint forces do not affect the markers' trajectories but only the bodies they are anchored to. Initially, markers are anchored at $\tilde{\boldsymbol{s}}_{ij} = \begin{bmatrix} 0 \\ 0 \\ 0 \end{bmatrix}$. This mapping attaches the marker to body b_j's center of mass.

This mapping is a very rough estimate of the marker attachment points on the model segments, but it is sufficient because of the flexible nature of constraints in the simulation software. Setting the *CFM* parameter of the marker constraints to $\beta = 10^{-3}$ and setting the model joint constraint *CFM* to $\beta = 10^{-5}$ makes the body segments hold together tightly while still allowing the markers to pull the body into shape. Several timesteps of simulation allow the model to relax to a fixed pose. We then take the markers in their current configuration and reattach them to their respective segments. Relaxing the marker attachments this way improves the fit for this particular frame of marker data. Iteratively repeating this process with multiple frames of marker data, we after that update the marker attachment points by some learning rate, η_m: $\tilde{\boldsymbol{s}}'_{ij} = (1 - \eta_m)\tilde{\boldsymbol{s}}_{ij} + \eta_m \boldsymbol{R}_j^{\mathrm{T}} (\boldsymbol{p}_i - \boldsymbol{x}_j)$. Gradually updating attachment points, using different frames of data, effectively descends the error gradient of the marker positions relative to the body:

$$\min_{\tilde{\boldsymbol{s}}} \sum_{t=1}^{T} \sum_{i=1}^{n_m} \|\boldsymbol{p}_i - \boldsymbol{R}_j \tilde{\boldsymbol{s}}_{ij} - \boldsymbol{x}_j\|$$

The decrease in marker error is affected by model dimension error. Conveniently, joint anchor constraints behave the same as the marker attachment constraints. With an arbitrary frame of marker data and using a marker *CFM* of $\beta = 10^{-4}$, if the markers constraints cannot be satisfied, they will pull the joint anchors apart slightly. We find a new common anchor point in the global frame for each joint by taking the average

between the two unsatisfied anchor points that the joint constraint is trying to pull together. We then move the anchor points toward that point according to learning rate η_l:

$$\tilde{\boldsymbol{c}}'_{jk} = (1-\eta_l)\tilde{\boldsymbol{c}}_{jk} + \eta_l \boldsymbol{R}_j^{\mathrm{T}} \left(\boldsymbol{R}_k \tilde{c}_{kj} + \boldsymbol{x}_k - \boldsymbol{x}_j\right)$$

For any frame, errors will cause the markers to stretch from their attachment points and joint anchor points to stretch apart from each other. Both marker attachment points and the joint anchors can be updated simultaneously to decrease the error for that frame. However, the local solution that perfectly satisfies one frame may make another frame worse. This step presents an evident gradient descent approach to finding the joint anchors and marker attachments: using several frames, compute an average adjustment to the marker attachments and joint anchors that reduce the error, make the adjustment to both anchors and attachments, and then iterate.

REFERENCES

Baraff, D. (1994). Fast contact force computation for nonpenetrating rigid bodies. In *Proceedings of the 21st annual conference on Computer graphics and interactive techniques*. 23–34

Baumgarte, J. (1972). Stabilization of constraints and integrals of motion in dynamical systems. *Computer methods in applied mechanics and engineering* 1, 1–16

Buss, S. R. (2000). Accurate and efficient simulation of rigid-body rotations. *Journal of Computational Physics* 164, 377–406

Catto, E. (2005). Iterative dynamics with temporal coherence. In *Game developer conference*. vol. 2

Featherstone, R. (2014). *Rigid body dynamics algorithms* (Springer)

Grassia, F. S. (1998). Practical parameterization of rotations using the exponential map. *Journal of graphics tools* 3, 29–48

Lanczos, C. (2020). *The variational principles of mechanics* (University of Toronto press)

Scholz, J. P. and Schöner, G. (1999). The uncontrolled manifold concept: identifying control variables for a functional task. *Experimental brain research* 126, 289–306

Smith, R. (2005). Constraints in rigid body dynamics. *Game Programming Gems* 4, 241–251

Smith, R. et al. (2005). Open dynamics engine
